# Genetic variations associated with six-white-point coat pigmentation in Diannan small-ear pigs

**DOI:** 10.1038/srep27534

**Published:** 2016-06-08

**Authors:** Meng-Die Lü, Xu-Man Han, Yun-Fei Ma, David M. Irwin, Yun Gao, Jia-Kun Deng, Adeniyi C. Adeola, Hai-Bing Xie, Ya-Ping Zhang

**Affiliations:** 1 State Key Laboratory of Genetic Resources and Evolution, Yunnan Laboratory of Molecular Biology of Domestic Animals, Kunming Institute of Zoology, Chinese Academy of Sciences, Kunming, China; 2 Kunming College of Life Science, University of Chinese Academy of Sciences, Kunming, China; 3 University of Chinese Academy of Sciences, Beijing, China; 4 Laboratory for Conservation and Utilization of Bio-Resources, Key Laboratory for Microbial Resources of the Ministry of Education, Yunnan University, Kunming, China

## Abstract

A common phenotypic difference among domestic animals is variation in coat color. Six-white-point is a pigmentation pattern observed in varying pig breeds, which seems to have evolved through several different mechanistic pathways. Herein, we re-sequenced whole genomes of 31 Diannan small-ear pigs from China and found that the six-white-point coat color in Diannan small-ear pigs is likely regulated by polygenic loci, rather than by the *MC1R* locus. Strong associations were observed at three loci (*EDNRB*, *CNTLN*, and *PINK1*), which explain about 20 percent of the total coat color variance in the Diannan small-ear pigs. We found a mutation that is highly differentiated between six-white-point and black Diannan small-ear pigs, which is located in a conserved noncoding sequence upstream of the *EDNRB* gene and is a putative binding site of the CEBPB protein. This study advances our understanding of coat color evolution in Diannan small-ear pigs and expands our traditional knowledge of coat color being a monogenic trait.

Domestication of the pig has been accompanied with retention of a tremendous amount of phenotypic variation regarding growth, behavior, and coat color. The diverse domestic pig breeds thus provide valuable animal models for genetic studies on phenotypic variation^1^,^2^,^3^.

Coat color was potentially exposed to artificial selection at an ancient time, dating back to about 5,000 years ago^4^. Variation in coat color may have important implications for pig breed development and conservation. Distinct coat colors have evolved in different pig breeds and may even have been used as visual characteristic to distinguish between breeds^5^. The most common coat colors found in domestic pigs include black, brown, white, red, spotted, belted, and two-end-black, which are distinct from the camouflage coat color of wild boars^4^,^6^,^7^,^8^,^9^. The genetic mechanisms underlying variation in coat color is an interesting topic.

Six-white-point (SWP) is a common kind of coat color in pigs that and is characterized by a white coat on the four feet, the head, and the end of tail but with the remaining coat being black. The SWP phenotype is observed in many well-known European and East Asian domestic pigs, including the Berkshire pigs from Europe^10^, as well as Diannan small-ear (DSE), Tibetan, Longlin and Anqing six-white-point pigs in China^11^.

The SWP coat color in the Berkshire pigs was found to be influenced by the melanocortin 1 receptor encoding (*MC1R*) gene, with this phenotype regarded as a special presentation of spotted color, as it is usually observed with black spots on white or red backgrounds^5^. In Berkshire pigs, two mutations in the *MC1R* gene are likely involved in formation of the SWP coat color pattern. The D124N *MC1R* allele probably causes a complete black body color^4^. SWP in the Berkshire is derived from the *MC1R* D124N allele through a 2-bp insertion in the coding region that leads to a coding frameshift and a premature stop codon. The background skin expresses the 2-bp inserted *MC1R* premature transcript, while the black-spotted area expresses a transcript without the 2-bp insertion, possibly due to somatic reversion events^10^. Additional to the *MC1R* locus, further evidence indicated that variation in the *KITLG* gene may also influence the coat color of Berkshire pigs^12^.

Due to the independent domestication of pigs in Europe and East Asia^13^,^14^, black coat color has putatively evolved through different genetic mutations in the coding sequence of the *MC1R* gene in East Asia (L102P) and Europe (D124N)^4^,^15^. Thus how the SWP phenotype evolved in East Asian domestic pigs, where these animals have a *MC1R* allele containing the L102P substitution remains mysterious.

In this study, we aimed to screen for genomic variants underlying the SWP phenotype using whole genome resequencing of DSE pigs from Yunnan province, China. DSE pigs have varying coat colors, including whole body black (denoted as “black” below) and SWP. Here we show that the SWP phenotype in DSE pigs is not attributed to variation in the *MC1R* gene, but rather it is associated with variations in multiple genes with differing phenotypic contributions.

## Results

### Coat colors in DSE pigs

**Figure 1:**
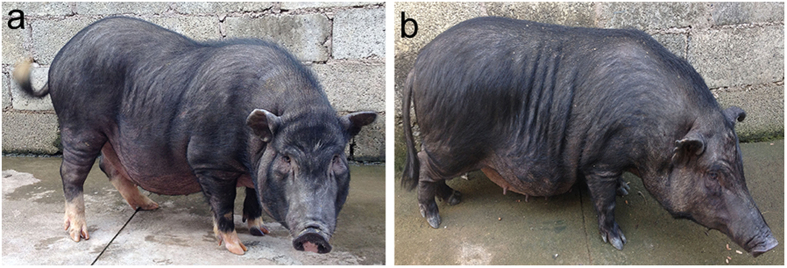
Typical coat color phenotypes in Diannan small-ear pigs. (**a**) six-white-point pigmentation. (**b**) whole body black pigmentation.

To obtain coat color phenotype data on the DSE pig, we collected pig samples and classified their coat color as either SWP or black. A total of 1,977 DSE pig samples were collected with coat color phenotype information with 753 (38%) of the DSE pigs being black and 1,224 (62%) being SWP. A comparison of whole black body and SWP phenotypes of DSE pigs is shown in Fig. 1. The white point on the head of the DSE pigs is located on the forehead. The SWP phenotype in the examined DSE individuals is highly variable, with 1,171 of the 1,224 SWP having a partial phenotype with the absence of the white coat at one or more of the six points. From our findings, black individuals were observed in about 78.6% of litters produced by SWP parents, and about 33% were black in the total population produced by SWP parents. SWP individuals were observed in about 25.5% of litters from black parents, and about 21.6% were SWP in the total population from black parents. About 47% SWP and 53% black offspring were observed in the cross between black and SWP DSE pigs.

### Whole genome resequencing of DSE pigs

To study the SWP phenotype in the DSE pigs, we selected 31 individuals (17 SWP and 14 black) for whole genome re-sequencing. Among the 17 SWP individuals, 11 had the typical SWP phenotype. Genomes were sequenced at about 7× depth of coverage for 8 SWP individuals and about 3× for the remaining 23 individuals.

Genomic reads were quality filtered and mapped to the pig reference genome^16^. On average, the resequencing data for each individual covered about 75% of the reference genome (Supplementary Table S1). We screened for genomic variants in the 31 individuals and a total of 23.9 million SNPs were identified. Among these SNPs, only about 150K and 107K were located in protein coding and UTR sequences, respectively, while most of them were located in noncoding sequences (6M in introns and 17.5M in intergenic sequences). Further classification showed that 69,707 SNPs were located in conserved sequences, 2,717,284 in putative regulatory sequences, and 347,597 in putative transcription factor DNA-binding motifs. Detailed information on the distribution of SNPs on each chromosome is shown in Supplementary Table S2.

### Analysis of the *MC1R* gene

To determine whether the SWP in the DSE pigs is associated with the 2-bp insertion in the coding sequence of the *MC1R* gene, as previously shown in Berkshire pigs^10^, we searched for the presence of a 2-bp insertion in *MC1R* in SWP and black DSE pigs. The 2-bp insertion in *MC1R* was not detected in any of the re-sequenced DSE pigs, and both the SWP and black DSE pigs were homozygous for the expected L102P *MC1R* allele (Supplementary Table S3). This observation implies that the SWP phenotype in the DSE pigs is not caused by a 2-bp insertion in the *MC1R* gene.

Black East Asian domestic pigs carry a L102P missense mutation in the *MC1R* gene^10^, as compared to a D124N mutation in some black domestic pigs from Europe^4^, and all of the re-sequenced DSE samples are homozygous for L102P. No mutations were identified at the D124 position (Supplementary Table S3), confirming that the *MC1R* allele in DSE pigs is of East Asian ancestry and not of European origin. In our comparative analysis, the *MC1R* locus does not show strong differentiation between the black and SWP DSE pigs (data not shown). These results further imply that the SWP coat color in DSE pigs likely is not be attributed by *MC1R* mutations.

### Screening for genomic regions putatively associated with SWP phenotype

**Figure 2:**
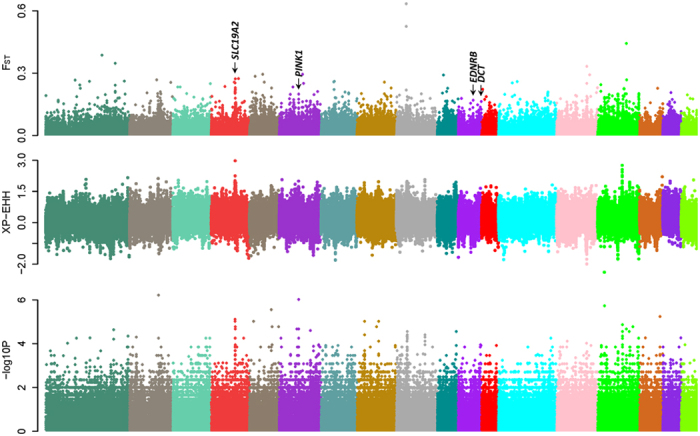
Whole genome screening of loci putatively associated with the SWP phenotype in DSE pigs. (**a**) Autosomal plot of genomic differentiation (F_ST_) between the SWP and black DSE pigs.(**b**) Autosomal XP-EHH plot in SWP DSE pigs with black DSE pigs as a background. (**c**) Association of IBD segments with the coat color phenotypes in the whole genome resequenced DSE pigs.

We first screened genomic regions that were highly differentiated between the SWP and black DSE pigs. In this analysis, 751 genes (Supplementary Table S4) were identified in genomic 10-kb sliding windows (containing at least 100 SNPs) with *F*_ST_ values above the chromosomal top 1% threshold (Fig. 2a, Supplementary Table S5). Some genes in the identified gene set are well known for roles in coat color regulation^17^, including the endothelin receptor type B (*EDNRB*) and dopachrome tautomerase (*DCT*) genes. *EDNRB* is important in the development of melanocytes^18^ and is involved in two-end-black phenotype regulation in pigs^12^,^19^,^20^. *DCT* null mice show diluted pigmentation with reduced melanin content in hair^21^.

To determine whether some haplotypes are associated with the SWP phenotype in DSE pigs, we compared the extended haplotype homozygosity (EHH) between SWP and whole black DSE pigs with an XP-EHH analysis. In addition, as SWP DSE pigs may share some genomic sequences that were inherited from a recent common ancestor, we also examined the inheritance of genomic sequences by analyzing the sharing of identity-by-descent (IBD) segments in the DSE pigs.

The XP-EHH- and IBD-based screening approaches (Fig. 2b,c) identified several genomic regions (Supplementary Table S6) replicating the signals in the *F*_ST_ analysis at the PTEN induced putative kinase 1 (*PINK1*) locus on chromosome 6, and at the *SLC19A2* gene on chromosome 4.

### Association analysis of candidate genes in a large population of DSE pigs

**Figure 3:**
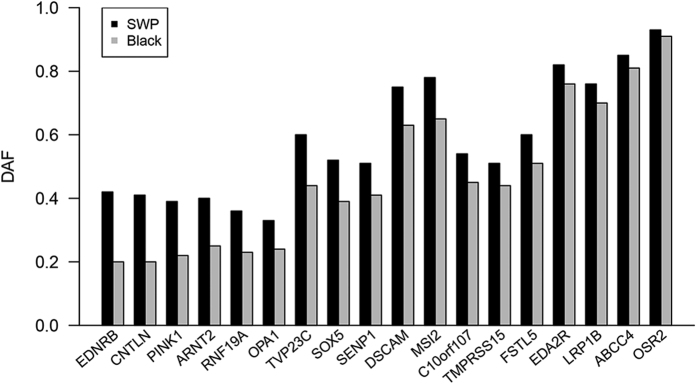
Derived allele frequency distribution of 18 tagged SNPs in 384 DSE pigs. The 18 SNPs represent 18 distinct loci. The allele frequencies were calculated in both the SWP (n = 169) and black (n = 215) DSE pigs.

To further analyze the association of SWP with candidate genes identified in our analysis, we focused on 18 SNPs, representing 18 different candidate genes (Supplementary Table S7). These 18 SNPs were chosen in genomic regions (<1Mb) showing a clustering of at least twenty 10-kb sliding windows with *F*_ST_ above the chromosomal top 10% threshold and containing at least three 10-kb sliding windows (each containing at least 100 SNPs) with window *F*_ST_ values above the chromosomal top 5% threshold. We selected the 18 SNPs with a rationale that each was from the top 10 SNPs having the highest *F*_ST_ values in each region, with a preference for a SNP covered by the largest number of resequenced individuals and avoiding SNPs located in repetitive sequences. A large population (n = 384) of DSE pigs with their coat color phenotype information was collected, where 215 were black and 169 were SWP pigs (37.87% of which had the typical SWP phenotype). The genotypes of all 18 SNPs were determined from all 384 individuals. Genotyping showed that the derived allele frequency difference between the SWP and black DSE populations were in concordance with that obtained in the whole genome re-sequenced population (Fig. 3). Of the 18 SNPs, 3 SNPs showed a statistically significant (*P* < 0.05; Pearson’s Chi-squared test) difference in their allele frequencies between the black and SWP individuals. However, none of the SNPs were completely fixed in the SWP pigs.

The association of the genotypes at the 18 SNPs and the SWP phenotype in the 384 phenotypic pigs was tested and unveiled an association with the SWP phenotype for 11 of these SNPs. Pronounced associations were observed at 3 SNPs from the genomic regions containing the *EDNRB* (*P* = 1.01 × 10^−11^; OR = 2.988), centlein, centrosomal protein (*CNTLN*) (*P* = 5.58 × 10^−10^; OR = 2.714), and *PINK1* (*P* = 3.04 × 10^−7^; OR = 2.269) encoding genes. Additional associations were observed for eight SNPs linked to the Golgi apparatus membrane protein TVP23 homolog C (*TVP23C*) (*P* = 1.12 × 10^−5^; OR = 1.908), aryl hydrocarbon receptor nuclear translocator 2 (*ARNT2*) (*P* = 1.37 × 10^−5^; OR = 1.983), ring finger protein 19A, E3 ubiquitin protein ligase (*RNF19*) (*P* = 1.15 × 10^−4^; OR = 1.857), coiled-coil domain containing 182 (*MSI2*) (*P* = 1.80 × 10^−4^; OR = 1.851), Down Syndrome cell adhesion molecule (*DSCAM*) (*P* = 3.41 × 10^−4^; OR = 1.775), SRY-box 5 (*SOX5*) (*P* = 2.04 × 10^−4^; OR = 1.725), sentrin-specific protease 1 (*SENP1*) (*P* = 3.0 × 10^−3^; OR = 1.544), and optic atrophy 1 (*OPA1*) (*P* = 3.66 × 10^−3^; OR = 1.597) encoding genes.

In a least-squares linear regression analysis, we found that genetic variation in *EDNRB*, *CNTLN*, and *PINK1* genes individually explain about 12.68%, 9.36%, and 7.14% coat color phenotypic variance in the DSE pigs respectively. However, the fraction of phenotypic variance explained by a single locus might be overestimated due to neglecting the fraction of variance explained by other loci, conditional on the genotypes at the focused locus. We performed a multiple regression analysis with the genetic variation combinations from these three loci, which showed that 18.52% of the coat color phenotypic variation was explained by the genetic co-variation from the *EDNRB* + *CNTLN* loci. Similarly, combinations of *EDNRB* + *PINK1* and *CNTLN* + *PINK1* explained 17.11% and 11.49% of the phenotypic variance, respectively. Genetic variation from the three loci explains 19.98% of the total phenotypic variance. The genetic variation from all 18 SNPs explains about 31.35% of the total phenotypic variance.

### Potential regulatory sequence mutations in the *EDNRB* locus

We are interested in associations between the *EDNRB* gene and the SWP phenotype. Earlier studies revealed that *EDNRB* is an important gene in pigmentation regulation and is putatively involved in the two-end-black pigmentation in pigs^12^,^19^. In our analysis, the *EDNRB* locus showed the strongest association with the SWP phenotype and explained the largest fraction of the phenotypic variance.

We screened the SNPs at the *EDNRB* locus to identify mutations that are highly differentiated between the SWP and black DSE pigs. In the whole genome re-sequenced DSE population, a total of 452 SNPs were identified, of which 179 (Supplementary Table S8) were identified with differentiation between the SWP and black pigs above the chromosomal top 10% threshold. These 179 SNPs were all located in the intergenic sequence upstream of the *EDRNB* gene (Supplementary Figure S1).

In a 100-way cross-species whole genome alignment analysis, 12 of the 179 SNPs (Table 1) were located in conserved noncoding sequences (CNS). As CNSs are a class of noncoding sequences that have evolved under strong evolutionary constraint, they are considered to potentially influence gene expression^22^. The location of SNPs inside a CNS is indicative of functional changes to expressional regulation of the *EDRNB* gene.

**Figure 4:**
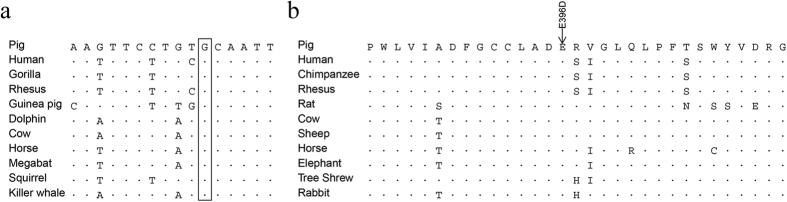
Cross-species alignment of genomic sequences enclosing two SNPs in the *EDNRB* and *PINK1* loci. (**a**) A SNP (chr11: 54,769,424; *G>A*) in the CEBPB DNA-binding motif upstream of the *EDNRB* gene. The position of the mutation position is indicated by a box. (**b**) A nonsynonymous mutation in the kinase domain of the PINK1. The arrow indicates the position of the mutation at a highly conserved residue from a variety of mammals.

Interestingly, a SNP (*G>A*; chr11: 54,769,424) is located in a sequence which is orthologous to the human counterpart that has been demonstrated to be a DNA-binding motif for the CCAAT/enhancer binding protein, beta (CEBPB), a b-ZIP transcription factor that binds to the consensus sequence (5′-T[TG]NNGNAA[TG]-3′) in promoters and upstream elements and stimulates gene expression^23^. A cross-species genome alignment showed that this CEBPB DNA-binding motif upstream of the *EDNRB* gene is highly conserved in mammals (Fig. 4a), indicating that it likely has an important biological function under strong evolutionary constraint. Strikingly, the *G>A* SNP position is centered at the binding motif in the CEBPB binding sequence and the mutation alters the DNA-binding sequence to 5′-T[TG]NNANAA[TG]-3′ (underlined “A” is the derived allele in the SWP DSE pigs), at a nucleotide that is highly conserved in the enhancers of a variety of genes^23^. This mutation may lead to a change in transactivation efficiency. In the whole genome resequencing, the *G>A* SNP was detected in reads from 12 SWP and 13 whole body black DSE pigs. Among the 12 SWP pigs, 10 were homozygous for the derived allele (adenine), and 2 were heterozygous, while in comparison, in the 13 black pigs, 6 were homozygous for the ancestral allele, 5 were heterozygous, and only 2 were homozygous for the derived allele. However, this SNP is likely in linkage with, rather than being, a causative mutation, inferring from the distribution of mutant homozygotes in the black DSE pigs.

Besides the SNP at the CEBPB recognition site, 11 additional SNPs (Table 1) were located in putative regulatory sequences. Among these, 3 SNPs (chr11: 54,769,314, 54,769,363, and 54,769,400) were found in the CEBPB binding sequence, with two SNPs (chr11: 54,770,500 and 54,770,533) identified in a sequence orthologous to a human sequence detected as a binding site by chromatin immunoprecipitation (ChIP) with the transcription factors STAT3, BACH1, FOS, and MAFK. Two additional SNPs (chr11: 54,769,730 and 54,769,742) were located in sequences orthologous to a human BACH1 binding sequence and a DNase I hypersensitive site. The SNP data set provides important mutation candidates for the SWP phenotype study.

### Mutation analysis of the *PINK1* locus

A 280-kb genomic sequence on chromosome 6 showed *F*_ST_ values reaching the chromosomal top 5% threshold. A total of 2,140 SNPs were found in this region. Among them, 553 SNPs were identified with *F*_ST_ values above the chromosomal top 10%. Six protein coding genes, including *CAMK2N1*, *MUL1*, *FAM43B*, *CDA*, *PINK1*, and *DDOST*, were identified within this 280-kb genomic span. The peak of the *F*_ST_ values of the 10-kb sliding windows is located on the *PINK1* gene.

Screening the 553 SNPs revealed two nonsynonymous and two synonymous mutations that were differentiated between the black and SWP DSE pigs. The two nonsynonymous mutations alter two residues (P195S and E396D) that are located in the kinase domain. The *F*_ST_ values are 0.42 and 0.24 at the P195S (chr6: 73,118,955; C>T) and E396D (chr6: 73,125,086; G>C) mutation sites. However, the SWP DSE pigs show an ancestral allele frequency (0.76) higher than the black DSE pigs (0.06) at the P195S coding site. In comparison, the derived allele frequency in the SWP DSE pigs (0.95) is much higher than in the black DSE pigs (0.40) at the E396D coding site. A multi-species sequence alignment showed that residue E396 is highly conserved in vertebrates (Fig. 4b), indicating a functional importance. Mutations at highly conserved residues in the kinase domain have been reported to impair or eliminate the PINK1 function^24^, thus the nonsynonymous mutation E396D potentially influences PINK1 function.

## Discussion

Selection for coat color has occurred since the domestication of the pig. Substantial variation in coat color exists in domestic pigs, and this phenotype has been a focus of evolutionary studies^25^. In this study, we examined the genetics underlying the SWP phenotype in DSE pigs, a well-known indigenous domestic pig from China.

The SWP phenotype is highly variable in the DSE pigs and with a complicated mode of inheritance. Our findings showed that black parents have SWP offspring while SWP parents black offspring. Therefore, we inferred from our observations that based on the occurrence of the SWP coat color in DSE pigs, this phenotype is likely to be under the regulation of polygenic loci. If the SWP was caused by a monogenic mutation, there should not be an occurrence of SWP parents with black offspring when SWP phenotype was inherited in a recessive mode, or alternatively, there should not be an occurrence of black parents with SWP offspring when SWP phenotype was inherited in a dominant mode. The inheritance of SWP phenotype is consistent with the multifactorial inheritance model of discrete traits proposed by Fisher^26^. However, we failed to identify an exact model for this phenotype inheritance. The higher proportion of black individuals observed as compared to the SWP individuals from a cross between black and SWP DSE pigs indicates a recessive mode of SWP inheritance at most of the associated loci. The association of the SWP phenotype with multiple loci is an indication of a complicated regulatory mechanism. Complete co-segregation of SWP with any mutation from a single locus in the DSE pigs could not be found, instead, many loci showed moderate levels of allele frequency differences in the SWP and black DSE populations. This suggests that different loci have varying contributions to the formation of the SWP phenotype. Furthermore, an increased fraction of the phenotypic variance can be explained by the combination of multiple loci compared to any individual locus. This is in sharp contrast to the black coat color that is likely under the regulation of missense mutations in the single *MC1R* gene in East Asian domestic pigs and wild boars^4^,^15^.

Our results support a role of *EDNRB* in the regulation of the SWP phenotype. *EDNRB* encodes a G-protein-coupled heptahelical receptor, for the ligand endothelin-3 (EDN3), which is an important signaling pathway for establishing melanocyte development^18^. Mutations in *EDNRB* have been widely reported to affect the pigmentation in a variety of organisms, including mice^27^,^28^,^29^, horse^30^,^31^, zebrafish^32^, and human^33^. It is interesting to observe the strong association of the *EDNRB* gene with the SWP phenotype in DSE pigs, as variation in *EDNRB* is also associated with the two-end-black pigmentation pattern seen in many different Chinese indigenous pig breeds^34^. In contrast, we found that the SWP phenotype is probably regulated by mutations in the upstream regulatory sequences of the *EDNRB* gene. The abundance of regulatory sequences inside and surrounding the *EDNRB* genes may partially explain the reason for the involvement of this locus in the regulation of several different coat color phenotypes. We infer that mutations in these putative *EDNRB* regulatory sequences might have altered tempospatial gene expression of downstream target genes. Consistently, *EDNRB* shows a temporal expression pattern^18^ and regulatory sequence variation results in altered pigmentation^35^.

The observed association of the *PINK1* locus with SWP implies a role of melanocyte death in SWP regulation. *PINK1* is widely accepted as a predisposition gene for early onset Parkinson disease^36^,^37^. Germline deletion of *PINK1* in mice significantly impaired the mitochondrial function^38^. RNA interference-induced *PINK1* deficiency causes mitochondrial accumulation of calcium, impaired mitochondrial respiration, and ultimately cell death^39^. The early onset of Parkinson disease may be caused by *PINK1*-mediated selective death of nerve cells. We propose that the SWP phenotype in some DSE individuals is attributed to *PINK1*-mediated selective death of melanocytes. This speculation is supported by our observation that, in some SWP DSE pigs, the area of white coat on four legs can increase, but never decrease, as a SWP DSE pig ages (data not shown), a phenomenon that can be explained by an irreversible melanocyte death process.

Besides these well-known loci, our study identified several new candidate genes for further study of coat color phenotypic variation in pigs, although the underlying mechanism is not well understood. The putative role of these genes should be tested with functional experiments.

This study provided us an opportunity to detect genetic loci associated with the SWP phenotype in DSE pigs and enrich our understanding on pigmentation regulation in domestic pigs. The results expanded our knowledge on coat color regulation, and provided additional insight on the well-addressed monogenic pigmentation regulation mechanisms and should have further implications on the conservation of the DSE pig.

## Methods

All methods were performed in accordance with the guidelines approved by the Kunming Institute of Zoology, Chinese Academy of Sciences. All experimental protocols were approved by the Kunming Institute of Zoology, Chinese Academy of Sciences.

### Sample and phenotype information collection

Samples and their phenotypes were collected from a purebred DSE pig population from our farm in Xishuangbanna, Yunnan Province, China. We collected DSE pig samples at day 1 after birth with a record of their coat color. All collected individuals were denoted as either black or SWP, irrespective of the moderate level of variations regarding the area, presence/absence of the white spots in some points in the SWP individuals. The occurrence of the SWP coat color was counted from 1,977 DSE pigs and 415 DSE pigs (186 SWP and 229 black) were used in this study.

### Whole genome resequencing

17 SWP and 14 black DSE pigs were included in the whole genome resequencing. Genomic DNA was extracted using the phenol-chloroform method and precipitated by 75% alcohol. Genomic DNA resequencing libraries were constructed with an insert size of 500-bp, following the Illumina library protocols. 100-bp paired-end reads were generated from the resequencing libraries with a Hiseq2000 instrument (Illumina Inc.). 8 SWP samples were sequenced to a coverage depth of about 7× and the other samples to about 3×.

### Variant calling and SNP annotation

The genomic reads were aligned to the pig reference genome (*Sus Scrofa* 10.2)^16^ with the BWA program^40^. Alignment outputs (BAM files) were sorted and PCR duplicates generated during genomic library construction were removed using SAMtools^41^. Genomic variants were called using the GATK^42^.

SNPs were annotated with the GTF file downloaded from the Ensembl website. In the annotation, a SNP was classified into protein coding (synonymous or nonsynonymous), untranslated region, intron, or intergenic according to their genomic position in the protein coding genes. To further annotate the SNPs in putative regulatory sequences, we downloaded human data from the Encyclopedia of DNA elements (ENCODE)^43^ project and identified putative regulatory sequences in the pig genome that are orthologous to the human counterpart with an ENCODE annotation (manuscript in preparation). Conserved noncoding sequences were identified from a 100-way whole genome alignment from 100 vertebrates that was downloaded from the UCSC genome browser (URL: http://genome.ucsc.edu). SNPs were analyzed according to their locations in the putative regulatory sequences and conserved noncoding sequences.

### Identification of putative loci underlying SWP regulation

Genetic differentiation (*F*_ST_) between SWP and black DSE pigs was calculated using VCFtools^44^, with 10-kb non-overlapping sliding windows for the autosomes. Genes in the sliding windows with *F*_ST_ above the chromosomal top 1% threshold were used as candidates for the SWP phenotype. Haplotypes were inferred using Beagle software^45^. XP-EHH was calculated to examine haplotypes that showed low levels of linkage decay in the SWP DSE pigs^46^,^47^,^48^. To examine inheritance from a common ancestor, identity-by-descendent (IBD) segments between any two individuals were identified using the GERMLINE program^49^, and the clustering of IBD segments was calculated using the DASH program^50^. The association between IBD clusters and the SWP phenotype was calculated using the PLINK software^51^.

### Genotyping and association analysis

To further analyze the association of candidate loci with the SWP phenotypes in a large DSE population, the genotypes of 18 tagged SNPs was established from a total of 384 DSE pigs (215 black and 169 SWP) using the Sequenom MassArray platform (Agena Bioscience). The derived allele was deduced as alternative allele to that in the pig reference genome. The 18 SNPs were chosen as tags for 18 loci that were putatively associated with the SWP phenotypes. SNPs and primers are shown in Table 2. Associations were calculated with the PLINK software^51^. The fraction of phenotypic variance explained by each locus was calculated as the R^2^ value in a least-squares linear regression analysis with a model


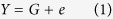


where Y is the coat color phenotype, G is the fixed effects of genotyped SNPs, and e is residual error.

## Additional Information

**How to cite this article**: Lü, M.-D. *et al*. Genetic variations associated with six-white-point coat pigmentation in Diannan small-ear pigs. *Sci. Rep*. **6**, 27534; doi: 10.1038/srep27534 (2016).

## Supplementary Material

Supplementary Information

## Figures and Tables

**Table 1:** SNPs located in putative regulatory sequences at the *EDNRB* locus on pig chromosome 11.

Position	Alleles	DNaseI	TFBS	TFBS Motif	CNS	Pig sequence (chr11)	Human sequence (chr13)
54,766,623	C/T	Yes	–	–	Yes	54,766,521–54,766,671	78,543,585–78,543,735
54,769,314	A/T	–	CEBPB	–	Yes	54,769,309–54,769,517	78,553,924–78,554,189
54,769,363	G/A	–	CEBPB	–	Yes	54,769,309–54,769,517	78,553,924–78,554,189
54,769,400	G/A	–	CEBPB	–	Yes	54,769,309–54,769,517	78,553,924–78,554,189
54,769,424	G/A	–	CEBPB	CEBPB	Yes	54,769,416–54,769,431	78,554,033–78,554,048
54,769,730	T/C	Yes	BACH1	–	Yes	54,769,711–54,769,791	78,554,376–78,554,463
54,769,742	C/T	Yes	BACH1	–	Yes	54,769,711–54,769,791	78,554,376–78,554,463
54,770,500	C/A	Yes	STAT3;BACH1;FOS;MAFK	–	Yes	54,770,363–54,770,597	78,554,832–78,555,068
54,770,533	G/A	Yes	STAT3;BACH1;FOS;MAFK	–	Yes	54,770,363–54,770,597	78,554,832–78,555,068
54,832,568	C/T	Yes	–	–	Yes	54,832,544–54,832,572	78,561,160–78,561,188
54,837,948	A/G	–	–	–	Yes	54,837,930–54,837,965	78,565,249–78,565,284
54,837,959	A/G	–	–	–	Yes	54,837,930–54,837,965	78,565,249–78,565,284

DNaseI indicates a DNase I hypersensitive site; TFBS indicates transcription factor binding site; TFBS Motif indicates a transcription factor DNA-binding motif; CNS indicates a conserved noncoding sequence.

**Table 2:** Primer information for 18 candidate SNPs used in the genotyping of DSE pigs.

CHR	POSITION	Forward PCR Primer	Reverse PCR Primer	Site-specific extension primer
chr13	140,229,419	ACGTTGGATGGGAGAACTTCATGGATCCTG	ACGTTGGATGGCCTACACTTAAAGATATTC	TACTATTATAGATATAACCTGCCATTTT
chr15	11,815,900	ACGTTGGATGGATATAGACTCTGACCAACC	ACGTTGGATGGCAAATCTCTTCTTAACAG	CCCTCTTCTTAACAGTTTTAATTAGG
chr15	92,243,930	ACGTTGGATGGGTGAGCTGCATTGTAAGAG	ACGTTGGATGCTAGTGTGATCAGCATGTCG	CAGTGCAATATTCAAAAGTC
chr6	73,118,955	ACGTTGGATGGGAATGATCTCTGGGCCTC	ACGTTGGATGTATGAGGCCACCATGCCTGT	AGCACCAGGCTTCTT
chr1	229,151,265	ACGTTGGATGGCATCACATGTATTAATAG	ACGTTGGATGCAGCTCTTGATACTGAGAAC	GAGAACATTTACCATATATCTTTTAT
chr4	40,901,362	ACGTTGGATGCTTGAGACATGGCAACAGAG	ACGTTGGATGAGGTAGAAAGACAGAGCCAG	TCCAACAAACACCTTTTT
chr4	39,532,637	ACGTTGGATGTCAGTAGTCAAAAGAAGCCG	ACGTTGGATGGTCTGTCTTTTGATGCTATCC	GCTATCCATATTTTAATATCTGC
chr14	70,198,568	ACGTTGGATGGTCACTTGACAGAGTTTCAC	ACGTTGGATGGATAGGGCTGTATTCCAAAC	AATGCTGTCATACTATATGATGTAA
chr5	53,130,878	ACGTTGGATGGTATGTCCTCTGTCCTCTTC	ACGTTGGATGCATCATGTTACGCTCCACAG	GGCTCCACAGAATAGTGTTTAATAAG
chr7	54,807,944	ACGTTGGATGTGTTGTGGGACTTAGAGGTG	ACGTTGGATGCATAGAAACAAAACCTAGTG	GGAACAAAACCTAGTGTAGTCAA
chr8	53,930,374	ACGTTGGATGGCGAATCAACATAAAAAAGG	ACGTTGGATGTCCCAAAGGTGATAGAAAAG	ATGTAGAAAGATTTATAGGGC
chr12	34,761,833	ACGTTGGATGTGCCTGTCTCAGAAAGCATC	ACGTTGGATGCAGAGAAGTGTCGGAACATC	CGGAACATCTTTGTAATGA
chr13	193,328,223	ACGTTGGATGGTGCCCAACAAAAGGCTATC	ACGTTGGATGTGAACTTCAAGTTTTTCCC	CTTTTTTCCCATTTTTAGAACATAC
chr13	213,659,304	ACGTTGGATGCAGCGTACTGATTTCCCTTC	ACGTTGGATGTCACGTTCCTTCCCCTACAG	TCCCGTGACTGTTGCACAC
chr5	81,642,632	ACGTTGGATGCATCGGATACACGTATCTTA	ACGTTGGATGCTTGGAATCCATTCTCCTGC	GGACTCCATTCTCCTGCTTGTCTA
chr11	71,412,807	ACGTTGGATGGACTGTTAGTATAGATTTCAG	ACGTTGGATGAAACCACTGCTGGTTGTTG	CCCGATTTCCCACAGCAATAA
chr11	54,762,085	ACGTTGGATGCAGACCCTTTTGTGAAAGGC	ACGTTGGATGATTTCCAACACCTGCAAGCC	AAGCCAATAATCTGATTTTTCC
chr11	81,925,800	ACGTTGGATGGAGAATAACATGAATGTTGGC	ACGTTGGATGACAACACCGAAATGAACCAG	CCAGAAAACTGAAAGGG
