# Supplementary material for: Genetic variations associated with six-white-point coat pigmentation in Diannan small-ear pigs
Source: Sci Rep. 2016 Jun 8;6:27534. doi: 10.1038/srep27534 (PMC4897638; doi:10.1038/srep27534)
Supplement: Supplementary Information [file srep27534-s1.doc]

**Supplementary Information for**

**Genetic variations associated with six-white-point coat pigmentation in Diannan small-ear pigs**

Meng-Die Lü^1,2,3†^, Xu-Man Han^1†^, Yun-Fei Ma^1^, David M. Irwin^1^, Yun Gao^1^, Jia-Kun Deng^1^, Adeniyi C. Adeola^1,2^, Hai-Bing Xie^1*^, Ya-Ping Zhang^1,4*^

^1^ State Key Laboratory of Genetic Resources and Evolution, Yunnan Laboratory of Molecular Biology of Domestic Animals, Kunming Institute of Zoology, Chinese Academy of Sciences, Kunming, China

^2^ Kunming College of Life Science, University of Chinese Academy of Sciences, Kunming, China

^3^ University of Chinese Academy of Sciences, Beijing, China

^4^ Laboratory for Conservation and Utilization of Bio-Resources, Key Laboratory for Microbial Resources of the Ministry of Education, Yunnan University, Kunming, China

† These authors contributed equally to this work.

* Author for Correspondence: Dr. Hai-Bing Xie ([xiehb@mail.kiz.ac.cn](mailto:xiehb@mail.kiz.ac.cn)) and Dr. Ya-Ping Zhang ([zhangyp@mail.kiz.ac.cn](mailto:zhangyp@mail.kiz.ac.cn))

**Table of contents**

**Supplementary Figure S1**

Supplementary Figure S1 | Distribution of 179 differentiated SNPs on the upstream of the *EDNRB* gene on pig chromosome 11.

**Supplementary Tables S1-S8**

Supplementary Table S1 | Information on individuals used in this study and their genome resequencing data statistics.

Supplementary Table S2 | Distribution and annotation of SNPs identified in this study.

Supplementary Table S3 | Information on mutation sites in *MC1R* that were reported in East Asian and European domestic pigs, as well as pigs with spotted pigmentation.

Supplementary Table S4 | List of 751 highly differentiated genes identified in the comparison of black and SWP DSE pigs.

Supplementary Table S5 | List of 10-kb sliding windows (containing at least 100 SNPs) highly differentiated in the black and SWP DSE pigs.

Supplementary Table S6 | Genomic signals identified by XP-EHH- and IBD-based screening of SWP regulating loci.

Supplementary Table S7 | 18 tagged SNPs for 18 candidate genes used in an association analysis of the SWP phenotype.

Supplementary Table S8 | SNPs upstream of the *EDNRB* on chromosome 11 highly differentiated between SWP and black DSE pigs.

**Supplementary Figure S1 | Distribution of 179 differentiated SNPs on the upstream of the *EDNRB* gene on pig chromosome 11.**

**
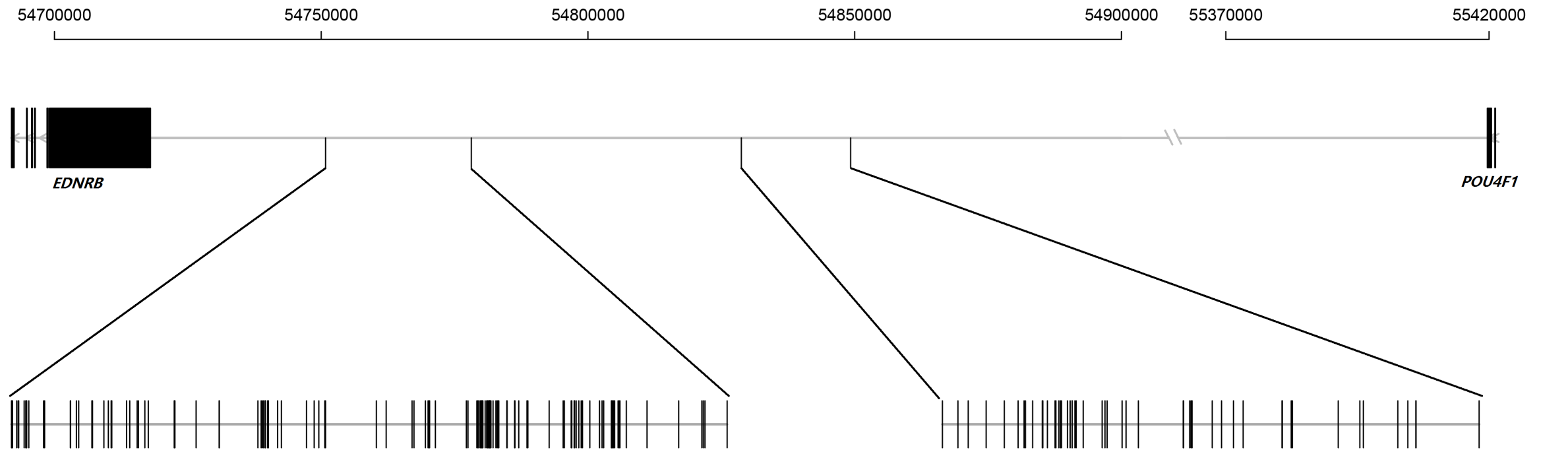
**

**Supplementary Table S1 | Information on individuals used in this study and their genome resequencing data statistics.**

| Population | Sample ID | Total reads | Unmapped reads | Mapped reads | Genome coverage |
| --- | --- | --- | --- | --- | --- |
| Six-white-point | 1 | 2,006,304,820 | 15,809,887 | 1,990,494,933 | 76.66% |
|  | 2 | 1,953,633,729 | 22,036,730 | 1,931,596,999 | 74.39% |
|  | 3 | 2,002,641,730 | 17,462,306 | 1,985,179,424 | 76.45% |
|  | 4 | 2,011,481,645 | 17,284,936 | 1,994,196,709 | 76.80% |
|  | 5 | 1,980,577,961 | 18,563,508 | 1,962,014,453 | 75.56% |
|  | 6 | 1,943,058,106 | 21,171,777 | 1,921,886,329 | 74.01% |
|  | 7 | 2,022,280,731 | 9,577,390 | 2,012,703,341 | 77.51% |
|  | 8 | 2,017,478,023 | 9,708,420 | 2,007,769,603 | 77.32% |
|  | 9 | 2,017,594,338 | 9,789,691 | 2,007,804,647 | 77.32% |
|  | 10 | 2,018,858,197 | 10,038,993 | 2,008,819,204 | 77.36% |
|  | 11 | 2,024,862,948 | 9,336,021 | 2,015,526,927 | 77.62% |
|  | 12 | 2,027,138,624 | 8,821,379 | 2,018,317,245 | 77.73% |
|  | 13 | 2,021,192,316 | 9,543,915 | 2,011,648,401 | 77.47% |
|  | 14 | 2,017,167,644 | 10,162,278 | 2,007,005,366 | 77.29% |
| Whole body black | 15 | 1,961,485,354 | 20,452,813 | 1,941,032,541 | 74.75% |
|  | 16 | 1,996,719,510 | 24,200,258 | 1,972,519,252 | 75.96% |
|  | 17 | 1,805,868,333 | 54,655,563 | 1,751,212,770 | 67.44% |
|  | 18 | 1,985,832,112 | 18,242,323 | 1,967,589,789 | 75.77% |
|  | 19 | 1,929,621,781 | 25,758,635 | 1,903,863,146 | 73.32% |
|  | 20 | 1,962,839,819 | 29,893,253 | 1,932,946,566 | 74.44% |
|  | 21 | 1,875,949,117 | 24,043,419 | 1,851,905,698 | 71.32% |
|  | 22 | 1,938,757,619 | 27,050,362 | 1,911,707,257 | 73.62% |
|  | 23 | 1,855,644,343 | 27,168,350 | 1,828,475,993 | 70.42% |
|  | 24 | 1,986,922,776 | 16,554,282 | 1,970,368,494 | 75.88% |
|  | 25 | 1,950,967,243 | 19,398,224 | 1,931,569,019 | 74.39% |
|  | 26 | 1,974,232,891 | 16,169,425 | 1,958,063,466 | 75.41% |
|  | 27 | 1,867,923,953 | 21,955,892 | 1,845,968,061 | 71.09% |
|  | 28 | 1,958,411,087 | 26,215,724 | 1,932,195,363 | 74.41% |
|  | 29 | 2,010,714,611 | 14,284,331 | 1,996,430,280 | 76.89% |
|  | 30 | 1,946,950,567 | 28,016,870 | 1,918,933,697 | 73.90% |
|  | 31 | 1,995,476,172 | 22,233,374 | 1,973,242,798 | 75.99% |

**Supplementary Table S2 | Distribution and annotation of SNPs identified in this study.**

| CHR | Total SNP | Conserved domain | ENCODE | Motif | Intergenic region | Intron | UTR | CDS |
| --- | --- | --- | --- | --- | --- | --- | --- | --- |
| Chr1 | 2,332,801 | 7,318 | 299,794 | 41,105 | 1,668,779 | 636,687 | 9,809 | 13,984 |
| Chr2 | 1,620,879 | 5,206 | 181,136 | 23,695 | 1,191,391 | 402,633 | 8,661 | 19,226 |
| Chr3 | 1,475,716 | 3,914 | 151,519 | 17,719 | 1,079,000 | 371,655 | 8,106 | 9,765 |
| Chr4 | 1,400,253 | 4,780 | 194,538 | 24,863 | 1,052,762 | 335,217 | 5,864 | 7,408 |
| Chr5 | 1,154,630 | 2,690 | 110,096 | 13,528 | 781,182 | 347,934 | 5,789 | 8,924 |
| Chr6 | 1,466,488 | 4,511 | 168,410 | 23,855 | 1,069,404 | 375,952 | 8,466 | 10,050 |
| Chr7 | 1,501,606 | 6,236 | 227,696 | 26,426 | 1,084,531 | 394,615 | 8,206 | 12,673 |
| Chr8 | 1,420,374 | 2,443 | 105,725 | 15,315 | 1,073,856 | 336,769 | 4,831 | 5,425 |
| Chr9 | 1,613,819 | 3,992 | 174,921 | 21,729 | 1,166,955 | 427,546 | 7,203 | 12,334 |
| Chr10 | 1,067,888 | 2,369 | 94,347 | 12,912 | 820,382 | 236,905 | 3,502 | 3,882 |
| Chr11 | 995,699 | 1,834 | 88,861 | 13,999 | 791,600 | 197,183 | 2,398 | 3,172 |
| Chr12 | 758,359 | 3,387 | 105,818 | 11,757 | 490,024 | 253,649 | 6,346 | 9,336 |
| Chr13 | 1,780,360 | 3,100 | 138,793 | 19,017 | 1,359,505 | 401,898 | 6,327 | 8,342 |
| Chr14 | 1,451,081 | 6,080 | 234,684 | 26,534 | 995,871 | 438,643 | 7,149 | 9,019 |
| Chr15 | 1,396,700 | 4,227 | 139,303 | 21,009 | 1,070,591 | 315,851 | 4,300 | 6,213 |
| Chr16 | 945,303 | 2,256 | 99,790 | 12,419 | 744,620 | 194,311 | 2,789 | 3,038 |
| Chr17 | 808,771 | 3,060 | 111,405 | 10,484 | 578,598 | 214,633 | 4,134 | 4,501 |
| Chr18 | 718,445 | 2,304 | 90,448 | 11,231 | 497,777 | 214,182 | 3,178 | 3,511 |

ENCODE indicate that the SNPs that were located in sequences of human counterparts having ENCODE annotations. Motif indicates that the SNPs were located in transcription factor DNA-binding motif sequences of human counterparts.

**Supplementary Table S3 | Information on mutation sites in *MC1R* that were reported in East Asian and European domestic pigs, as well as pigs with spotted pigmentation.**

| Sample ID | 6C/8C | L102P | D124N |
| --- | --- | --- | --- |
| 1 | 6C | P | D |
| 2 | 6C | P | D |
| 3 | 6C | P | D |
| 4 | 6C | P | D |
| 5 | 6C | P | D |
| 6 | 6C | P | D |
| 7 | 6C | P | D |
| 8 | 6C | P | D |
| 9 | 6C | P | D |
| 10 | 6C | P | D |
| 11 | 6C | P | D |
| 12 | 6C | P | D |
| 13 | 6C | P | D |
| 14 | N/A | P | D |
| 15 | 6C | P | D |
| 16 | 6C | P | D |
| 17 | 6C | P | D |
| 18 | 6C | P | D |
| 19 | 6C | P | D |
| 20 | N/A | P | D |
| 21 | 6C | P | D |
| 22 | 6C | P | N/A |
| 23 | 6C | P | D |
| 24 | 6C | P | D |
| 25 | 6C | P | D |
| 26 | 6C | P | D |
| 27 | 6C | P | D |
| 28 | 6C | P | D |
| 29 | 6C | P | D |
| 30 | 6C | P | D |
| 31 | 6C | P | D |

**Supplementary Table S4 | List of 751 highly differentiated genes identified in the comparison of black and SWP DSE pigs**.

| Chromosome | Genes |
| --- | --- |
| 1 | *ADAMTSL1; ANXA1; C6ORF118; C6ORF170; C6ORF211; C6ORF97; C9ORF46; C9orf57; CEP78; CNTLN; DCC; DNAJC25; ENSSSCG00000004665; ENSSSCG00000005198; ENSSSCG00000005223; ENSSSCG00000005391; ENSSSCG00000005721; ENSSSCG00000021072; ENSSSCG00000022532; ENSSSCG00000022796; ENSSSCG00000024501; ENSSSCG00000024731; ENSSSCG00000024876; ENSSSCG00000026182; ENSSSCG00000026988; ENSSSCG00000027081; ENSSSCG00000028037; ENSSSCG00000028987; EPHA7; FIG4; GABRA5; GCNT1; GDA; GLIS3; GNA14; KTN1; MAN1A1; MAPK4; MDGA2; MED27; MRO; MUSK; NPR2; OR1J2; OSTF1; PCSK5; PELI2; PTGR1; QKI; RFK; RGP1; RORB; RPL10L; RPS29; RSPH3; SH3GL2; SNX30; SQRDL; SVEP1; TLE1; UNC13C* |
| 2 | *ANO5; C5ORF13; C5orf24; DPF2; ENSSSCG00000013186; ENSSSCG00000013400; ENSSSCG00000014176; ENSSSCG00000014178; ENSSSCG00000014403; ENSSSCG00000021549; ENSSSCG00000029033; ENSSSCG00000029860; EPB41L4A; FAM174A; FER; INSC; IRAP; LIX1; LRRC4C; MAN2A1; MRPL16; MSH3; NELL1; NR3C1; OR4K13; PARVA; PPP2R2B; REEP5; RIOK2; SEC24A; SEMA6A; SOX6; STARD4; STX3; TMEM232; TXNDC15* |
| 3 | *ASAP2; ASB3; C1D; C2orf49; CALN1; CCDC85A; CMPK2; COMMD1; CRIM1; ENSSSCG00000007725; ENSSSCG00000007729; ENSSSCG00000007735; ENSSSCG00000007760; ENSSSCG00000007899; ENSSSCG00000007919; ENSSSCG00000008250; ENSSSCG00000008418; ENSSSCG00000008469; ENSSSCG00000021334; ENSSSCG00000023060; ENSSSCG00000023401; ENSSSCG00000024602; ENSSSCG00000025072; ENSSSCG00000025164; ENSSSCG00000025751; ENSSSCG00000026180; ENSSSCG00000028309; ENSSSCG00000028743; ENSSSCG00000029209; ENSSSCG00000030195; ENSSSCG00000030512; ETAA1; FHL2; FOXN2; FSHR; GALM; GPR45; KAT8; LLGL1; MAP4K4; MBOAT2; MYT1L; NCK2; NUP54; PABPC4; PARN; RBFOX1; RDH14; RFX8; SMC6; SOX11; SRCAP; SUCLG1; SULT1C3; TAF1B; TGFBRAP1; TMEM114; TMEM121; TSSC1; ZCWPW1* |
| 4 | *AMY2B; ATP1B1; ATP6V1H; BLZF1; C1ORF110; C1orf114; C20ORF132; CA2; COL11A1; CRP; DDR2; DPT; DUSP23; ENSSSCG00000006139; ENSSSCG00000006253; ENSSSCG00000006254; ENSSSCG00000006320; ENSSSCG00000021936; F5; FAM78B; GPA33; ILDR2; NIPAL2; NME7; OPRK1; PGCP; POGK; TADA1; TBX19; TMCO1; XCL1; ZNF696* |
| 5 | *ABCC9; AEBP2; BCAT1; C12orf63; CAPRIN2; CAPZA3; CDK17; CMAS; ENSSSCG00000000506; ENSSSCG00000000565; ENSSSCG00000000747; ENSSSCG00000000818; ENSSSCG00000021948; ENSSSCG00000023758; ENSSSCG00000026193; ENSSSCG00000027447; ENSSSCG00000027682; ETV6; H1FNT; IQSEC3; ITPR2; LDOC1L; LGR5; NEDD1; OR10AD1; PARVB; PDE9A; PFKM; RECQL; RERGL; RFX4; TMPO; TRHDE; ZNF641* |
| 6 | *ALG6; ANKRD27; AQP4; ATG4C; C1ORF141; C1orf168; C1ORF87; CACHD1; CALB2; CDA; CDH2; CDYL2; CELF4; CMIP; CMPK1; DPY19L3; EFCAB7; ENSSSCG00000002691; ENSSSCG00000003676; ENSSSCG00000003725; ENSSSCG00000003824; ENSSSCG00000003838; ENSSSCG00000003871; ENSSSCG00000023243; ENSSSCG00000024494; ENSSSCG00000024963; ENSSSCG00000029984; FAM43B; FPGT; GAN; GLIS1; KCTD1; LRRIQ3; MUL1; NEGR1; OSBPL9; PDCD5; PIK3C3; PINK1; PLA2G2D; PLA2G2F; PLA2G5; PLCG2; PRKAA2; RAB3B; RPE65; SLC35D1; TCEANC2; TM2D1; TMEM48; TNNI3K; TXNDC12; UBE2U; WLS; ZFYVE9; ZNF260; ZNF382; ZNF529* |
| 7 | *ADAMTSL3; ANKRD34C; ARNT2; ARRDC4; ATXN1; AVEN; C6ORF132; C6ORF62; CD2AP; CLEC14A; CRISP1; DEFB110; DEFB133; DNAL1; E2F3; EFTUD1; EML5; ENSSSCG00000001458; ENSSSCG00000001729; ENSSSCG00000002045; ENSSSCG00000002253; ENSSSCG00000002255; ENSSSCG00000002256; ENSSSCG00000021507; ENSSSCG00000021722; ENSSSCG00000023038; ENSSSCG00000026114; ENSSSCG00000026127; ENSSSCG00000026370; FAH; FAM108C1; FCF1; FLRT2; FOXP4; GMDS; GPR110; ID4; INSM2; KIF6; LRFN2; MBOAT1; MDFI; MEP1A; NFKBIA; NFYA; NOVA1; PLEKHG3; PPP1R36; PRKD1; RASGRF1; RBM24; RNF144B; SEC23A; SLC25A27; STXBP6; TFAP2D; TNFRSF21; TRERF1; UBR2; UNC5CL* |
| 8 | *ARAP2; AREG; ATP5I; BTC; C4ORF33; C4orf51; CCRN4L; DCLK2; DNAJB14; ENOPH1; ENSSSCG00000008763; ENSSSCG00000008764; ENSSSCG00000008767; ENSSSCG00000008826; ENSSSCG00000008827; ENSSSCG00000009021; ENSSSCG00000009032; ENSSSCG00000009041; ENSSSCG00000009248; ENSSSCG00000022556; ENSSSCG00000027713; FAM47E; GABRA2; GABRA4; GABRG1; GUCY1B3; H2AFZ; HNRNPD; HNRPDL; METTL14; PDGFC; SCARB2; SCD5; SCLT1; SEC24D; TEC* |
| 9 | *ALKBH8; ANGPTL5; AQP11; ARHGAP20; C1ORF27; CADM1; CASP1; CASP12; CD3D; CD3E; CD3G; CDHR3; CDK14; CHORDC1; CNTN5; CWF19L2; ENSSSCG00000015055; ENSSSCG00000015064; ENSSSCG00000015242; ENSSSCG00000015434; ENSSSCG00000015531; ENSSSCG00000015553; ENSSSCG00000015571; ENSSSCG00000021012; ENSSSCG00000021545; ENSSSCG00000023029; ENSSSCG00000025517; ENSSSCG00000026049; ENSSSCG00000029504; FAT3; GRIA4; INTS4; IVNS1ABP; MACC1; MMP1; MMP10; NEU3; NPL; OR2AT4; PAK1; PDC; PDGFD; REXO2; RGS16; RNASEL; SEMA3E; SLC36A4; SP4; SP8; STEAP2; THRSP; TMEM196; TRPC6; UBE4A; ZNF804B* |
| 10 | *ADIPOR1; AGTPBP1; ARHGAP21; C9orf102; C9orf103; CTSL1; DAPK1; ENSSSCG00000011007; ENSSSCG00000021915; ENSSSCG00000022856; ENSSSCG00000026278; ENSSSCG00000026405; ENSSSCG00000028135; ENSSSCG00000028447; ENSSSCG00000029816; EPC1; FBP1; FBP2; FRMD3; GAS1; GOLM1; KIF5B; MPP7; MRPL51; NEBL; PIP4K2A; PPP1R12B; PTCH1; RMI1; SLC28A3; SPAG6; TRKB; UBQLN1; ZCCHC6; ZNF782* |
| 11 | *ALG11; ARGLU1; CCDC70; COG6; COL4A1; COL4A2; EDNRB; EFHA1; EFNB2; ENSSSCG00000009455; ENSSSCG00000009475; ENSSSCG00000016840; ENSSSCG00000021980; ENSSSCG00000026317; ENSSSCG00000029214; ENSSSCG00000029465; ENSSSCG00000030057; FAM155A; FGF14; FGF9; FOXO1A; GPC6; HTR2A; KATNAL1; KLHL1; POU4F1; RFC3; RPL5; SACS; SAP18; SLC25A15; SLITRK5; STARD13; SUCLA2; UBAC2; USPL1; ZC3H13; ZDHHC20* |
| 12 | *ABR; BHLHA9; BZRAP1; C17ORF57; ENSSSCG00000017303; ENSSSCG00000017597; ENSSSCG00000017598; ENSSSCG00000017619; ENSSSCG00000017620; ENSSSCG00000017675; ENSSSCG00000017712; ENSSSCG00000017892; ENSSSCG00000021572; ENSSSCG00000029475; MAP2K6; MARCH10; MMD; MPO; MRPS23; NLK; NSRP1; PLCD3; SOX9; TANC2; TBX4; TMEM100; TMEM95; TNK1* |
| 13 | *ALCAM; ASTE1; ATP2C1; B3GALNT1; BACE2; CADM2; CAMP; CPNE4; CXADR; DCBLD2; DSCAM; ENSSSCG00000011228; ENSSSCG00000011823; ENSSSCG00000012003; ENSSSCG00000012009; ENSSSCG00000012067; ENSSSCG00000021991; ENSSSCG00000022789; ENSSSCG00000022949; ENSSSCG00000025997; ENSSSCG00000026112; ENSSSCG00000027041; ENSSSCG00000028774; ENSSSCG00000029127; ENSSSCG00000029384; ENSSSCG00000030335; ENSSSCG00000030440; EPHA6; GADL1; GBE1; HLCS; IL20RB; KALRN; KCNJ15; KCNJ6; KLHL6; LAMP3; LIPI; MCM2; NCAM2; NEK11; NMD3; NSUN3; OPA1; PROK2; PSMG1; PXR; ROBO1; ROPN1; RTP1; RUNX1; RYBP; SETD4; SLC25A36; SOX14; SPINK8; SST; TMPRSS15; TRIM42; TTC21A; VEPH1; ZPLD1* |
| 14 | *ABCC2; ACTN2; ANKRD1; ANUBL1; B3GALNT2; BTAF1; CPEB3; CTNNA3; ENSSSCG00000009707; ENSSSCG00000010463; ENSSSCG00000021805; ENSSSCG00000023638; ENSSSCG00000025877; ENSSSCG00000028393; ENSSSCG00000029274; ENSSSCG00000029700; ENSSSCG00000030026; FBXO21; GALNT7; HTR7; KIF13B; KIF20B; KSR2; MBL2; MED13L; MSRA; MTR; NFIL3; NOS1; PCDH15; PCGF5; PIWIL3; PPP1R3C; PTEN; RFC5; RNLS; ROR2; TBX3; TNKS2; XPNPEP1* |
| 15 | *AGFG1; ANGPT2; CLN8; CWC22; DDX18; DPP10; ENSSSCG00000015750; ENSSSCG00000015786; ENSSSCG00000015839; ENSSSCG00000016006; ENSSSCG00000016347; ENSSSCG00000020860; ENSSSCG00000022603; ENSSSCG00000023419; ENSSSCG00000023746; ENSSSCG00000024217; ENSSSCG00000026504; ENSSSCG00000028949; HJURP; ITGA4; LRP1B; MCPH1; MLF1IP; NDUFA10; OSBPL6; PLEKHA2; PRKRA; STAT4; TM4SF20; TRPM8; WRN; ZNF385B; ZNF804A* |
| 16 | *ADAMTS16; ANKH; C1QTNF3; CCNG1; CLINT1; EBF1; EGFLAM; ENSSSCG00000016795; ENSSSCG00000016797; ENSSSCG00000017064; ENSSSCG00000017110; ENSSSCG00000017122; ENSSSCG00000020913; ENSSSCG00000025115; ENSSSCG00000025965; ENSSSCG00000026842; ENSSSCG00000027191; ENSSSCG00000027636; FBXL7; GABRG2; GPBP1; MED10; MRPL22; NMUR2; ORF; PAPD7; PLK2; PRLR; RAI14; SLIT3; SPEF2* |
| 17 | *ANKRD5; AVP; BMP2; BPIFB2; BPIFB3; BPIFB4; BTBD3; C8ORF79; CST7; EDN3; ENSSSCG00000007113; ENSSSCG00000007258; ENSSSCG00000007361; ENSSSCG00000007461; ENSSSCG00000007537; ENSSSCG00000021368; ENSSSCG00000028573; FLRT3; KIF16B; LONRF1; OTOR; OXT; PAK7; PAX1; PHACTR3; POLR3F; PTPRT; SPTLC3; SYNDIG1; TPT1; XRN2* |
| 18 | *BMPER; C7ORF25; CALD1; CHN2; CPVL; ENSSSCG00000016537; ENSSSCG00000016762; ENSSSCG00000024471; ENSSSCG00000026023; ENSSSCG00000027468; ENSSSCG00000028280; FAM3C; JHDM1D; KCND2; MRPL32; NPSR1; PARP12; PPP1R17; PTPRZ1; TTC26; VPS41* |

**Supplementary Table S5 | List of 10-kb sliding windows (containing at least 100 SNPs) highly differentiated in the black and SWP DSE pigs.**

| Chromosome | Start Position | End Position | Mean *F*_ST_ |
| --- | --- | --- | --- |
| 1 | 4910001 | 4920000 | 0.109196 |
| 1 | 10260001 | 10270000 | 0.08377 |
| 1 | 17160001 | 17170000 | 0.091699 |
| 1 | 45680001 | 45690000 | 0.093925 |
| 1 | 45990001 | 46000000 | 0.112411 |
| 1 | 66410001 | 66420000 | 0.090769 |
| 1 | 67170001 | 67180000 | 0.078995 |
| 1 | 85530001 | 85540000 | 0.082085 |
| 1 | 105710001 | 105720000 | 0.083407 |
| 1 | 110120001 | 110130000 | 0.092888 |
| 1 | 110170001 | 110180000 | 0.114697 |
| 1 | 110210001 | 110220000 | 0.131506 |
| 1 | 112990001 | 113000000 | 0.104948 |
| 1 | 113060001 | 113070000 | 0.099094 |
| 1 | 113070001 | 113080000 | 0.098446 |
| 1 | 130280001 | 130290000 | 0.08191 |
| 1 | 140670001 | 140680000 | 0.125374 |
| 1 | 156950001 | 156960000 | 0.087474 |
| 1 | 156960001 | 156970000 | 0.104347 |
| 1 | 196020001 | 196030000 | 0.093869 |
| 1 | 198840001 | 198850000 | 0.097759 |
| 1 | 205710001 | 205720000 | 0.099947 |
| 1 | 228030001 | 228040000 | 0.086129 |
| 1 | 229010001 | 229020000 | 0.114404 |
| 1 | 229420001 | 229430000 | 0.166729 |
| 1 | 240000001 | 240010000 | 0.089242 |
| 1 | 242430001 | 242440000 | 0.103544 |
| 1 | 242440001 | 242450000 | 0.111066 |
| 1 | 242460001 | 242470000 | 0.093694 |
| 1 | 243690001 | 243700000 | 0.091411 |
| 1 | 243700001 | 243710000 | 0.084625 |
| 1 | 251920001 | 251930000 | 0.081201 |
| 1 | 253290001 | 253300000 | 0.116121 |
| 1 | 253300001 | 253310000 | 0.083812 |
| 1 | 255480001 | 255490000 | 0.090248 |
| 1 | 256150001 | 256160000 | 0.081432 |
| 1 | 256160001 | 256170000 | 0.102393 |
| 1 | 257340001 | 257350000 | 0.078888 |
| 1 | 260440001 | 260450000 | 0.09278 |
| 1 | 260480001 | 260490000 | 0.131995 |
| 1 | 260490001 | 260500000 | 0.108199 |
| 1 | 260670001 | 260680000 | 0.083531 |
| 1 | 260720001 | 260730000 | 0.102996 |
| 1 | 264200001 | 264210000 | 0.080432 |
| 1 | 270630001 | 270640000 | 0.080026 |
| 1 | 282150001 | 282160000 | 0.080618 |
| 1 | 282180001 | 282190000 | 0.081245 |
| 1 | 282200001 | 282210000 | 0.079248 |
| 1 | 282500001 | 282510000 | 0.091131 |
| 1 | 283190001 | 283200000 | 0.09402 |
| 1 | 284330001 | 284340000 | 0.085905 |
| 1 | 296220001 | 296230000 | 0.080285 |
| 1 | 296610001 | 296620000 | 0.086382 |
| 1 | 305950001 | 305960000 | 0.083246 |
| 2 | 6010001 | 6020000 | 0.096848 |
| 2 | 11080001 | 11090000 | 0.097277 |
| 2 | 13200001 | 13210000 | 0.093675 |
| 2 | 24790001 | 24800000 | 0.093517 |
| 2 | 40910001 | 40920000 | 0.087005 |
| 2 | 46180001 | 46190000 | 0.099421 |
| 2 | 46220001 | 46230000 | 0.093509 |
| 2 | 50340001 | 50350000 | 0.095017 |
| 2 | 91120001 | 91130000 | 0.092992 |
| 2 | 107420001 | 107430000 | 0.086754 |
| 2 | 107430001 | 107440000 | 0.167239 |
| 2 | 107440001 | 107450000 | 0.138141 |
| 2 | 107450001 | 107460000 | 0.091732 |
| 2 | 107470001 | 107480000 | 0.091895 |
| 2 | 107480001 | 107490000 | 0.109536 |
| 2 | 108160001 | 108170000 | 0.144937 |
| 2 | 108170001 | 108180000 | 0.115235 |
| 2 | 108400001 | 108410000 | 0.098302 |
| 2 | 109930001 | 109940000 | 0.128099 |
| 2 | 110420001 | 110430000 | 0.147904 |
| 2 | 110430001 | 110440000 | 0.115977 |
| 2 | 110440001 | 110450000 | 0.147919 |
| 2 | 110450001 | 110460000 | 0.089469 |
| 2 | 110460001 | 110470000 | 0.086984 |
| 2 | 118330001 | 118340000 | 0.100303 |
| 2 | 118340001 | 118350000 | 0.143203 |
| 2 | 118410001 | 118420000 | 0.101839 |
| 2 | 119390001 | 119400000 | 0.097595 |
| 2 | 120880001 | 120890000 | 0.100596 |
| 2 | 121470001 | 121480000 | 0.085467 |
| 2 | 121780001 | 121790000 | 0.099038 |
| 2 | 122180001 | 122190000 | 0.113728 |
| 2 | 122230001 | 122240000 | 0.151447 |
| 2 | 122240001 | 122250000 | 0.0918 |
| 2 | 126150001 | 126160000 | 0.086575 |
| 2 | 126160001 | 126170000 | 0.094759 |
| 2 | 142520001 | 142530000 | 0.08636 |
| 2 | 142660001 | 142670000 | 0.113505 |
| 2 | 151470001 | 151480000 | 0.089912 |
| 2 | 154440001 | 154450000 | 0.10822 |
| 3 | 7670001 | 7680000 | 0.096409 |
| 3 | 12050001 | 12060000 | 0.119822 |
| 3 | 12060001 | 12070000 | 0.100282 |
| 3 | 12070001 | 12080000 | 0.093656 |
| 3 | 15740001 | 15750000 | 0.083654 |
| 3 | 16620001 | 16630000 | 0.087514 |
| 3 | 17500001 | 17510000 | 0.116428 |
| 3 | 18150001 | 18160000 | 0.088837 |
| 3 | 29840001 | 29850000 | 0.090092 |
| 3 | 32500001 | 32510000 | 0.105362 |
| 3 | 34990001 | 35000000 | 0.109357 |
| 3 | 37410001 | 37420000 | 0.101439 |
| 3 | 38340001 | 38350000 | 0.109883 |
| 3 | 50020001 | 50030000 | 0.093084 |
| 3 | 51600001 | 51610000 | 0.082964 |
| 3 | 51640001 | 51650000 | 0.200857 |
| 3 | 51650001 | 51660000 | 0.115784 |
| 3 | 51720001 | 51730000 | 0.090082 |
| 3 | 51840001 | 51850000 | 0.10316 |
| 3 | 55240001 | 55250000 | 0.121906 |
| 3 | 64190001 | 64200000 | 0.094395 |
| 3 | 68000001 | 68010000 | 0.119262 |
| 3 | 68020001 | 68030000 | 0.10299 |
| 3 | 77820001 | 77830000 | 0.108377 |
| 3 | 78240001 | 78250000 | 0.124579 |
| 3 | 82460001 | 82470000 | 0.111793 |
| 3 | 83570001 | 83580000 | 0.093477 |
| 3 | 88900001 | 88910000 | 0.090078 |
| 3 | 91510001 | 91520000 | 0.087069 |
| 3 | 94210001 | 94220000 | 0.096084 |
| 3 | 97460001 | 97470000 | 0.089087 |
| 3 | 98100001 | 98110000 | 0.083575 |
| 3 | 104610001 | 104620000 | 0.097645 |
| 3 | 105100001 | 105110000 | 0.090744 |
| 3 | 105330001 | 105340000 | 0.132531 |
| 3 | 105580001 | 105590000 | 0.116472 |
| 3 | 105590001 | 105600000 | 0.086547 |
| 3 | 107820001 | 107830000 | 0.119821 |
| 3 | 110820001 | 110830000 | 0.089564 |
| 3 | 111480001 | 111490000 | 0.140596 |
| 3 | 111930001 | 111940000 | 0.090266 |
| 3 | 111950001 | 111960000 | 0.08943 |
| 3 | 112640001 | 112650000 | 0.121036 |
| 3 | 127660001 | 127670000 | 0.09374 |
| 3 | 128270001 | 128280000 | 0.103668 |
| 3 | 134960001 | 134970000 | 0.103714 |
| 3 | 134970001 | 134980000 | 0.094237 |
| 3 | 135420001 | 135430000 | 0.090579 |
| 3 | 135430001 | 135440000 | 0.091076 |
| 3 | 138470001 | 138480000 | 0.085043 |
| 3 | 141210001 | 141220000 | 0.099047 |
| 4 | 1510001 | 1520000 | 0.122079 |
| 4 | 41520001 | 41530000 | 0.135425 |
| 4 | 41620001 | 41630000 | 0.123316 |
| 4 | 43020001 | 43030000 | 0.091481 |
| 4 | 56000001 | 56010000 | 0.092753 |
| 4 | 56020001 | 56030000 | 0.106169 |
| 4 | 83240001 | 83250000 | 0.098459 |
| 4 | 83250001 | 83260000 | 0.103113 |
| 4 | 83270001 | 83280000 | 0.098989 |
| 4 | 83280001 | 83290000 | 0.109493 |
| 4 | 84370001 | 84380000 | 0.097664 |
| 4 | 84380001 | 84390000 | 0.090809 |
| 4 | 84400001 | 84410000 | 0.103272 |
| 4 | 89060001 | 89070000 | 0.100809 |
| 4 | 89170001 | 89180000 | 0.121625 |
| 4 | 89240001 | 89250000 | 0.253355 |
| 4 | 89250001 | 89260000 | 0.212134 |
| 4 | 89260001 | 89270000 | 0.202666 |
| 4 | 89420001 | 89430000 | 0.112974 |
| 4 | 89440001 | 89450000 | 0.096344 |
| 4 | 89450001 | 89460000 | 0.10335 |
| 4 | 89520001 | 89530000 | 0.110901 |
| 4 | 89730001 | 89740000 | 0.2729 |
| 4 | 89890001 | 89900000 | 0.125249 |
| 4 | 89900001 | 89910000 | 0.155931 |
| 4 | 90160001 | 90170000 | 0.179395 |
| 4 | 91630001 | 91640000 | 0.095808 |
| 4 | 91830001 | 91840000 | 0.15005 |
| 4 | 91930001 | 91940000 | 0.10726 |
| 4 | 91940001 | 91950000 | 0.152456 |
| 4 | 91950001 | 91960000 | 0.110548 |
| 4 | 91960001 | 91970000 | 0.092961 |
| 4 | 91970001 | 91980000 | 0.146095 |
| 4 | 92020001 | 92030000 | 0.128332 |
| 4 | 92030001 | 92040000 | 0.102991 |
| 4 | 92100001 | 92110000 | 0.109954 |
| 4 | 92450001 | 92460000 | 0.102807 |
| 4 | 95470001 | 95480000 | 0.118941 |
| 4 | 98730001 | 98740000 | 0.107167 |
| 4 | 126380001 | 126390000 | 0.118387 |
| 4 | 127280001 | 127290000 | 0.091491 |
| 5 | 2030001 | 2040000 | 0.11425 |
| 5 | 13550001 | 13560000 | 0.116707 |
| 5 | 37890001 | 37900000 | 0.129798 |
| 5 | 40950001 | 40960000 | 0.11693 |
| 5 | 47090001 | 47100000 | 0.125021 |
| 5 | 50630001 | 50640000 | 0.117676 |
| 5 | 52690001 | 52700000 | 0.123735 |
| 5 | 52700001 | 52710000 | 0.113075 |
| 5 | 54720001 | 54730000 | 0.256938 |
| 5 | 54780001 | 54790000 | 0.166326 |
| 5 | 54790001 | 54800000 | 0.116658 |
| 5 | 54800001 | 54810000 | 0.127562 |
| 5 | 56740001 | 56750000 | 0.10885 |
| 5 | 57350001 | 57360000 | 0.140951 |
| 5 | 58190001 | 58200000 | 0.136504 |
| 5 | 63160001 | 63170000 | 0.125666 |
| 5 | 69720001 | 69730000 | 0.120267 |
| 5 | 72540001 | 72550000 | 0.128435 |
| 5 | 81680001 | 81690000 | 0.108451 |
| 5 | 81740001 | 81750000 | 0.114966 |
| 5 | 81800001 | 81810000 | 0.118594 |
| 5 | 81810001 | 81820000 | 0.185749 |
| 5 | 81820001 | 81830000 | 0.113479 |
| 5 | 81930001 | 81940000 | 0.136839 |
| 5 | 89730001 | 89740000 | 0.150379 |
| 5 | 91370001 | 91380000 | 0.106124 |
| 6 | 7170001 | 7180000 | 0.109657 |
| 6 | 7460001 | 7470000 | 0.098477 |
| 6 | 7480001 | 7490000 | 0.137006 |
| 6 | 8330001 | 8340000 | 0.15455 |
| 6 | 13910001 | 13920000 | 0.104964 |
| 6 | 37960001 | 37970000 | 0.100439 |
| 6 | 38020001 | 38030000 | 0.097176 |
| 6 | 41250001 | 41260000 | 0.157014 |
| 6 | 41260001 | 41270000 | 0.099193 |
| 6 | 41280001 | 41290000 | 0.121174 |
| 6 | 41290001 | 41300000 | 0.198094 |
| 6 | 41300001 | 41310000 | 0.126658 |
| 6 | 72580001 | 72590000 | 0.105418 |
| 6 | 72960001 | 72970000 | 0.174933 |
| 6 | 73030001 | 73040000 | 0.132642 |
| 6 | 73060001 | 73070000 | 0.19938 |
| 6 | 91500001 | 91510000 | 0.100065 |
| 6 | 104160001 | 104170000 | 0.102485 |
| 6 | 105550001 | 105560000 | 0.134622 |
| 6 | 116830001 | 116840000 | 0.106442 |
| 6 | 116840001 | 116850000 | 0.125194 |
| 6 | 116860001 | 116870000 | 0.110703 |
| 6 | 117270001 | 117280000 | 0.117834 |
| 6 | 126450001 | 126460000 | 0.113585 |
| 6 | 126520001 | 126530000 | 0.110735 |
| 6 | 128710001 | 128720000 | 0.111382 |
| 6 | 128840001 | 128850000 | 0.099246 |
| 6 | 128850001 | 128860000 | 0.10236 |
| 6 | 128880001 | 128890000 | 0.121042 |
| 6 | 128910001 | 128920000 | 0.127985 |
| 6 | 129040001 | 129050000 | 0.121228 |
| 6 | 129060001 | 129070000 | 0.114357 |
| 6 | 129070001 | 129080000 | 0.099126 |
| 6 | 130000001 | 130010000 | 0.147028 |
| 6 | 133570001 | 133580000 | 0.099324 |
| 6 | 133700001 | 133710000 | 0.138261 |
| 6 | 134400001 | 134410000 | 0.112329 |
| 6 | 136570001 | 136580000 | 0.098295 |
| 6 | 137270001 | 137280000 | 0.11956 |
| 6 | 137630001 | 137640000 | 0.108794 |
| 6 | 137640001 | 137650000 | 0.127506 |
| 6 | 137670001 | 137680000 | 0.124185 |
| 6 | 138730001 | 138740000 | 0.099707 |
| 6 | 138740001 | 138750000 | 0.101211 |
| 6 | 139760001 | 139770000 | 0.127175 |
| 6 | 139780001 | 139790000 | 0.108821 |
| 6 | 143490001 | 143500000 | 0.110585 |
| 6 | 143510001 | 143520000 | 0.108306 |
| 6 | 143550001 | 143560000 | 0.151273 |
| 6 | 143560001 | 143570000 | 0.162514 |
| 6 | 145940001 | 145950000 | 0.117148 |
| 6 | 146360001 | 146370000 | 0.100731 |
| 6 | 146370001 | 146380000 | 0.099473 |
| 6 | 146400001 | 146410000 | 0.105237 |
| 6 | 147680001 | 147690000 | 0.099966 |
| 6 | 147870001 | 147880000 | 0.110768 |
| 6 | 147880001 | 147890000 | 0.105335 |
| 6 | 148290001 | 148300000 | 0.11205 |
| 6 | 151730001 | 151740000 | 0.097337 |
| 7 | 1100001 | 1110000 | 0.101597 |
| 7 | 1190001 | 1200000 | 0.098497 |
| 7 | 13740001 | 13750000 | 0.101316 |
| 7 | 16020001 | 16030000 | 0.114236 |
| 7 | 16550001 | 16560000 | 0.109555 |
| 7 | 20660001 | 20670000 | 0.107283 |
| 7 | 29170001 | 29180000 | 0.117413 |
| 7 | 29180001 | 29190000 | 0.131636 |
| 7 | 40060001 | 40070000 | 0.107626 |
| 7 | 41380001 | 41390000 | 0.116572 |
| 7 | 41550001 | 41560000 | 0.113037 |
| 7 | 42080001 | 42090000 | 0.105427 |
| 7 | 42670001 | 42680000 | 0.126169 |
| 7 | 42840001 | 42850000 | 0.103709 |
| 7 | 42860001 | 42870000 | 0.099608 |
| 7 | 48100001 | 48110000 | 0.117766 |
| 7 | 48150001 | 48160000 | 0.099666 |
| 7 | 48250001 | 48260000 | 0.145269 |
| 7 | 48520001 | 48530000 | 0.118872 |
| 7 | 49070001 | 49080000 | 0.138299 |
| 7 | 49080001 | 49090000 | 0.11928 |
| 7 | 49170001 | 49180000 | 0.260198 |
| 7 | 49180001 | 49190000 | 0.110272 |
| 7 | 49230001 | 49240000 | 0.149489 |
| 7 | 49240001 | 49250000 | 0.09989 |
| 7 | 49250001 | 49260000 | 0.132914 |
| 7 | 49270001 | 49280000 | 0.110124 |
| 7 | 49380001 | 49390000 | 0.096581 |
| 7 | 49390001 | 49400000 | 0.102136 |
| 7 | 50300001 | 50310000 | 0.111403 |
| 7 | 50550001 | 50560000 | 0.120614 |
| 7 | 50800001 | 50810000 | 0.097589 |
| 7 | 53870001 | 53880000 | 0.099684 |
| 7 | 53910001 | 53920000 | 0.126203 |
| 7 | 53930001 | 53940000 | 0.097924 |
| 7 | 54800001 | 54810000 | 0.151163 |
| 7 | 54810001 | 54820000 | 0.111025 |
| 7 | 54830001 | 54840000 | 0.110527 |
| 7 | 56420001 | 56430000 | 0.105774 |
| 7 | 56490001 | 56500000 | 0.097704 |
| 7 | 65910001 | 65920000 | 0.09776 |
| 7 | 69380001 | 69390000 | 0.104658 |
| 7 | 75840001 | 75850000 | 0.111476 |
| 7 | 75850001 | 75860000 | 0.118451 |
| 7 | 78440001 | 78450000 | 0.112699 |
| 7 | 78450001 | 78460000 | 0.130502 |
| 7 | 81570001 | 81580000 | 0.102782 |
| 7 | 86480001 | 86490000 | 0.105509 |
| 7 | 88070001 | 88080000 | 0.116784 |
| 7 | 89540001 | 89550000 | 0.105616 |
| 7 | 95130001 | 95140000 | 0.120344 |
| 7 | 102710001 | 102720000 | 0.203119 |
| 7 | 103790001 | 103800000 | 0.103474 |
| 7 | 112870001 | 112880000 | 0.107275 |
| 7 | 117030001 | 117040000 | 0.101028 |
| 8 | 23780001 | 23790000 | 0.175142 |
| 8 | 27490001 | 27500000 | 0.100522 |
| 8 | 28770001 | 28780000 | 0.105474 |
| 8 | 29080001 | 29090000 | 0.105922 |
| 8 | 38140001 | 38150000 | 0.145992 |
| 8 | 38810001 | 38820000 | 0.101372 |
| 8 | 40160001 | 40170000 | 0.132317 |
| 8 | 40170001 | 40180000 | 0.100826 |
| 8 | 40590001 | 40600000 | 0.145608 |
| 8 | 47630001 | 47640000 | 0.124256 |
| 8 | 74740001 | 74750000 | 0.108801 |
| 8 | 76110001 | 76120000 | 0.096897 |
| 8 | 83360001 | 83370000 | 0.107489 |
| 8 | 83780001 | 83790000 | 0.11209 |
| 8 | 83790001 | 83800000 | 0.109758 |
| 8 | 88280001 | 88290000 | 0.095409 |
| 8 | 93940001 | 93950000 | 0.108412 |
| 8 | 102600001 | 102610000 | 0.11022 |
| 8 | 102620001 | 102630000 | 0.12904 |
| 8 | 102630001 | 102640000 | 0.114053 |
| 8 | 102670001 | 102680000 | 0.135891 |
| 8 | 112770001 | 112780000 | 0.101297 |
| 8 | 129770001 | 129780000 | 0.140932 |
| 8 | 129780001 | 129790000 | 0.101009 |
| 8 | 144980001 | 144990000 | 0.100246 |
| 8 | 145150001 | 145160000 | 0.109225 |
| 8 | 145160001 | 145170000 | 0.100696 |
| 8 | 145170001 | 145180000 | 0.10334 |
| 8 | 145180001 | 145190000 | 0.095336 |
| 8 | 145190001 | 145200000 | 0.121267 |
| 9 | 10430001 | 10440000 | 0.115254 |
| 9 | 10470001 | 10480000 | 0.095749 |
| 9 | 13030001 | 13040000 | 0.106551 |
| 9 | 13080001 | 13090000 | 0.096388 |
| 9 | 13170001 | 13180000 | 0.090064 |
| 9 | 13180001 | 13190000 | 0.114939 |
| 9 | 13720001 | 13730000 | 0.089728 |
| 9 | 13730001 | 13740000 | 0.088944 |
| 9 | 26490001 | 26500000 | 0.091399 |
| 9 | 28980001 | 28990000 | 0.097188 |
| 9 | 35350001 | 35360000 | 0.092778 |
| 9 | 36480001 | 36490000 | 0.123183 |
| 9 | 36520001 | 36530000 | 0.145663 |
| 9 | 37370001 | 37380000 | 0.096088 |
| 9 | 38800001 | 38810000 | 0.095525 |
| 9 | 38840001 | 38850000 | 0.097629 |
| 9 | 38970001 | 38980000 | 0.109209 |
| 9 | 39280001 | 39290000 | 0.093261 |
| 9 | 40010001 | 40020000 | 0.098813 |
| 9 | 40030001 | 40040000 | 0.09161 |
| 9 | 40060001 | 40070000 | 0.104119 |
| 9 | 40120001 | 40130000 | 0.098412 |
| 9 | 43200001 | 43210000 | 0.092151 |
| 9 | 46840001 | 46850000 | 0.091166 |
| 9 | 49020001 | 49030000 | 0.098379 |
| 9 | 49030001 | 49040000 | 0.097915 |
| 9 | 49040001 | 49050000 | 0.093079 |
| 9 | 49050001 | 49060000 | 0.133776 |
| 9 | 50680001 | 50690000 | 0.096489 |
| 9 | 50690001 | 50700000 | 0.089958 |
| 9 | 62810001 | 62820000 | 0.096745 |
| 9 | 76540001 | 76550000 | 0.099238 |
| 9 | 76790001 | 76800000 | 0.10132 |
| 9 | 76850001 | 76860000 | 0.122579 |
| 9 | 76860001 | 76870000 | 0.111908 |
| 9 | 76910001 | 76920000 | 0.097887 |
| 9 | 77430001 | 77440000 | 0.094612 |
| 9 | 77440001 | 77450000 | 0.100482 |
| 9 | 77470001 | 77480000 | 0.122367 |
| 9 | 77530001 | 77540000 | 0.10045 |
| 9 | 98180001 | 98190000 | 0.090424 |
| 9 | 99320001 | 99330000 | 0.095007 |
| 9 | 107040001 | 107050000 | 0.089071 |
| 9 | 116690001 | 116700000 | 0.090747 |
| 9 | 121690001 | 121700000 | 0.094482 |
| 9 | 133340001 | 133350000 | 0.130822 |
| 9 | 136200001 | 136210000 | 0.114773 |
| 9 | 136420001 | 136430000 | 0.095305 |
| 9 | 139370001 | 139380000 | 0.12969 |
| 9 | 140050001 | 140060000 | 0.095668 |
| 10 | 28870001 | 28880000 | 0.105205 |
| 10 | 28910001 | 28920000 | 0.120753 |
| 10 | 28950001 | 28960000 | 0.112339 |
| 10 | 28960001 | 28970000 | 0.141636 |
| 10 | 29270001 | 29280000 | 0.114033 |
| 10 | 30500001 | 30510000 | 0.102208 |
| 10 | 30680001 | 30690000 | 0.101279 |
| 10 | 30700001 | 30710000 | 0.101756 |
| 10 | 31220001 | 31230000 | 0.101213 |
| 10 | 31810001 | 31820000 | 0.106472 |
| 10 | 31880001 | 31890000 | 0.11555 |
| 10 | 31920001 | 31930000 | 0.100851 |
| 10 | 32140001 | 32150000 | 0.112723 |
| 10 | 32970001 | 32980000 | 0.138796 |
| 10 | 33230001 | 33240000 | 0.113362 |
| 10 | 33720001 | 33730000 | 0.103754 |
| 10 | 33970001 | 33980000 | 0.101526 |
| 10 | 33980001 | 33990000 | 0.11402 |
| 10 | 33990001 | 34000000 | 0.12415 |
| 10 | 34090001 | 34100000 | 0.105382 |
| 10 | 34430001 | 34440000 | 0.134281 |
| 10 | 34440001 | 34450000 | 0.116942 |
| 10 | 34770001 | 34780000 | 0.114607 |
| 10 | 34800001 | 34810000 | 0.109337 |
| 10 | 35410001 | 35420000 | 0.101532 |
| 10 | 35450001 | 35460000 | 0.106307 |
| 10 | 35470001 | 35480000 | 0.113676 |
| 10 | 35600001 | 35610000 | 0.132323 |
| 10 | 38730001 | 38740000 | 0.1026 |
| 10 | 38950001 | 38960000 | 0.101188 |
| 10 | 47370001 | 47380000 | 0.115625 |
| 10 | 52090001 | 52100000 | 0.122629 |
| 10 | 53040001 | 53050000 | 0.122998 |
| 10 | 55970001 | 55980000 | 0.114193 |
| 10 | 56080001 | 56090000 | 0.165266 |
| 10 | 58070001 | 58080000 | 0.103959 |
| 10 | 59660001 | 59670000 | 0.119676 |
| 10 | 76780001 | 76790000 | 0.120283 |
| 10 | 76790001 | 76800000 | 0.100188 |
| 11 | 410001 | 420000 | 0.092013 |
| 11 | 490001 | 500000 | 0.107881 |
| 11 | 510001 | 520000 | 0.085875 |
| 11 | 520001 | 530000 | 0.085651 |
| 11 | 530001 | 540000 | 0.084963 |
| 11 | 540001 | 550000 | 0.103466 |
| 11 | 580001 | 590000 | 0.087599 |
| 11 | 680001 | 690000 | 0.104132 |
| 11 | 1260001 | 1270000 | 0.102003 |
| 11 | 1450001 | 1460000 | 0.085975 |
| 11 | 6980001 | 6990000 | 0.111975 |
| 11 | 6990001 | 7000000 | 0.108764 |
| 11 | 9870001 | 9880000 | 0.095017 |
| 11 | 9880001 | 9890000 | 0.105292 |
| 11 | 11320001 | 11330000 | 0.177745 |
| 11 | 15220001 | 15230000 | 0.088222 |
| 11 | 15810001 | 15820000 | 0.091253 |
| 11 | 16310001 | 16320000 | 0.099792 |
| 11 | 20410001 | 20420000 | 0.116776 |
| 11 | 20570001 | 20580000 | 0.092013 |
| 11 | 21810001 | 21820000 | 0.102775 |
| 11 | 46010001 | 46020000 | 0.089356 |
| 11 | 54650001 | 54660000 | 0.088118 |
| 11 | 54750001 | 54760000 | 0.096748 |
| 11 | 54760001 | 54770000 | 0.134164 |
| 11 | 54830001 | 54840000 | 0.105498 |
| 11 | 55360001 | 55370000 | 0.087431 |
| 11 | 67880001 | 67890000 | 0.113014 |
| 11 | 67890001 | 67900000 | 0.094007 |
| 11 | 75380001 | 75390000 | 0.095411 |
| 11 | 75390001 | 75400000 | 0.117133 |
| 11 | 75400001 | 75410000 | 0.119057 |
| 11 | 75470001 | 75480000 | 0.121755 |
| 11 | 77820001 | 77830000 | 0.105854 |
| 11 | 79290001 | 79300000 | 0.095614 |
| 11 | 81180001 | 81190000 | 0.084357 |
| 11 | 81400001 | 81410000 | 0.12641 |
| 11 | 81730001 | 81740000 | 0.086223 |
| 11 | 81740001 | 81750000 | 0.090369 |
| 11 | 81820001 | 81830000 | 0.088613 |
| 11 | 81940001 | 81950000 | 0.136441 |
| 11 | 81950001 | 81960000 | 0.087896 |
| 11 | 81960001 | 81970000 | 0.089949 |
| 11 | 81970001 | 81980000 | 0.085879 |
| 11 | 82050001 | 82060000 | 0.085591 |
| 11 | 82530001 | 82540000 | 0.092002 |
| 11 | 82890001 | 82900000 | 0.103266 |
| 11 | 84460001 | 84470000 | 0.108241 |
| 11 | 84490001 | 84500000 | 0.108572 |
| 12 | 10460001 | 10470000 | 0.096761 |
| 12 | 10470001 | 10480000 | 0.172898 |
| 12 | 15870001 | 15880000 | 0.102918 |
| 12 | 16300001 | 16310000 | 0.091259 |
| 12 | 16430001 | 16440000 | 0.097216 |
| 12 | 18400001 | 18410000 | 0.088189 |
| 12 | 18530001 | 18540000 | 0.100022 |
| 12 | 29130001 | 29140000 | 0.093615 |
| 12 | 29250001 | 29260000 | 0.130586 |
| 12 | 29380001 | 29390000 | 0.14295 |
| 12 | 32640001 | 32650000 | 0.087295 |
| 12 | 34210001 | 34220000 | 0.140023 |
| 12 | 34230001 | 34240000 | 0.116447 |
| 12 | 34250001 | 34260000 | 0.096611 |
| 12 | 34740001 | 34750000 | 0.14005 |
| 12 | 34750001 | 34760000 | 0.135357 |
| 12 | 36240001 | 36250000 | 0.093435 |
| 12 | 38200001 | 38210000 | 0.092113 |
| 12 | 39330001 | 39340000 | 0.10535 |
| 12 | 41390001 | 41400000 | 0.091752 |
| 12 | 46290001 | 46300000 | 0.097853 |
| 12 | 48240001 | 48250000 | 0.12021 |
| 12 | 48250001 | 48260000 | 0.14721 |
| 12 | 48740001 | 48750000 | 0.110437 |
| 12 | 52870001 | 52880000 | 0.11426 |
| 12 | 54930001 | 54940000 | 0.107253 |
| 13 | 680001 | 690000 | 0.098576 |
| 13 | 930001 | 940000 | 0.096614 |
| 13 | 1070001 | 1080000 | 0.140074 |
| 13 | 1080001 | 1090000 | 0.099418 |
| 13 | 1090001 | 1100000 | 0.092844 |
| 13 | 1140001 | 1150000 | 0.098507 |
| 13 | 1180001 | 1190000 | 0.093066 |
| 13 | 19370001 | 19380000 | 0.090488 |
| 13 | 26070001 | 26080000 | 0.104577 |
| 13 | 34320001 | 34330000 | 0.112682 |
| 13 | 34330001 | 34340000 | 0.128902 |
| 13 | 58910001 | 58920000 | 0.092086 |
| 13 | 80190001 | 80200000 | 0.090481 |
| 13 | 80320001 | 80330000 | 0.096384 |
| 13 | 85740001 | 85750000 | 0.123973 |
| 13 | 89460001 | 89470000 | 0.088156 |
| 13 | 89470001 | 89480000 | 0.110678 |
| 13 | 105300001 | 105310000 | 0.123594 |
| 13 | 109180001 | 109190000 | 0.152962 |
| 13 | 109190001 | 109200000 | 0.116533 |
| 13 | 130700001 | 130710000 | 0.108399 |
| 13 | 130710001 | 130720000 | 0.118667 |
| 13 | 130720001 | 130730000 | 0.160606 |
| 13 | 134590001 | 134600000 | 0.151661 |
| 13 | 140190001 | 140200000 | 0.089143 |
| 13 | 140200001 | 140210000 | 0.115725 |
| 13 | 140220001 | 140230000 | 0.116237 |
| 13 | 145740001 | 145750000 | 0.134054 |
| 13 | 149760001 | 149770000 | 0.102765 |
| 13 | 157560001 | 157570000 | 0.112838 |
| 13 | 163950001 | 163960000 | 0.103101 |
| 13 | 163960001 | 163970000 | 0.113641 |
| 13 | 163970001 | 163980000 | 0.130449 |
| 13 | 170400001 | 170410000 | 0.119367 |
| 13 | 174430001 | 174440000 | 0.110213 |
| 13 | 174560001 | 174570000 | 0.109267 |
| 13 | 174600001 | 174610000 | 0.087253 |
| 13 | 181320001 | 181330000 | 0.097731 |
| 13 | 183820001 | 183830000 | 0.098672 |
| 13 | 184080001 | 184090000 | 0.112298 |
| 13 | 184340001 | 184350000 | 0.088549 |
| 13 | 184910001 | 184920000 | 0.08967 |
| 13 | 185220001 | 185230000 | 0.169208 |
| 13 | 185650001 | 185660000 | 0.120986 |
| 13 | 188800001 | 188810000 | 0.088482 |
| 13 | 191530001 | 191540000 | 0.098702 |
| 13 | 191730001 | 191740000 | 0.089934 |
| 13 | 193410001 | 193420000 | 0.15259 |
| 13 | 193430001 | 193440000 | 0.101732 |
| 13 | 193970001 | 193980000 | 0.096564 |
| 13 | 194780001 | 194790000 | 0.09363 |
| 13 | 208360001 | 208370000 | 0.157562 |
| 13 | 208380001 | 208390000 | 0.115859 |
| 13 | 210610001 | 210620000 | 0.103239 |
| 13 | 211690001 | 211700000 | 0.123182 |
| 13 | 212210001 | 212220000 | 0.09324 |
| 13 | 212220001 | 212230000 | 0.087885 |
| 13 | 212540001 | 212550000 | 0.144784 |
| 13 | 213690001 | 213700000 | 0.088166 |
| 13 | 213700001 | 213710000 | 0.114583 |
| 13 | 213800001 | 213810000 | 0.113447 |
| 13 | 213810001 | 213820000 | 0.090844 |
| 13 | 213920001 | 213930000 | 0.090243 |
| 13 | 214050001 | 214060000 | 0.088018 |
| 13 | 214410001 | 214420000 | 0.107006 |
| 13 | 214450001 | 214460000 | 0.115121 |
| 13 | 214500001 | 214510000 | 0.108934 |
| 13 | 214510001 | 214520000 | 0.127821 |
| 13 | 216530001 | 216540000 | 0.096001 |
| 14 | 3500001 | 3510000 | 0.090532 |
| 14 | 14140001 | 14150000 | 0.108695 |
| 14 | 14380001 | 14390000 | 0.101347 |
| 14 | 18050001 | 18060000 | 0.105042 |
| 14 | 19390001 | 19400000 | 0.093545 |
| 14 | 19530001 | 19540000 | 0.092045 |
| 14 | 19580001 | 19590000 | 0.117095 |
| 14 | 19660001 | 19670000 | 0.111462 |
| 14 | 36860001 | 36870000 | 0.090634 |
| 14 | 37450001 | 37460000 | 0.160349 |
| 14 | 37460001 | 37470000 | 0.095868 |
| 14 | 37500001 | 37510000 | 0.110963 |
| 14 | 37510001 | 37520000 | 0.10657 |
| 14 | 39960001 | 39970000 | 0.147642 |
| 14 | 39970001 | 39980000 | 0.094007 |
| 14 | 45370001 | 45380000 | 0.09796 |
| 14 | 58620001 | 58630000 | 0.093514 |
| 14 | 59880001 | 59890000 | 0.100692 |
| 14 | 73690001 | 73700000 | 0.106942 |
| 14 | 73700001 | 73710000 | 0.112682 |
| 14 | 73750001 | 73760000 | 0.155016 |
| 14 | 98700001 | 98710000 | 0.103797 |
| 14 | 98710001 | 98720000 | 0.09117 |
| 14 | 105050001 | 105060000 | 0.106267 |
| 14 | 105200001 | 105210000 | 0.099596 |
| 14 | 109290001 | 109300000 | 0.114018 |
| 14 | 110620001 | 110630000 | 0.098744 |
| 14 | 110630001 | 110640000 | 0.101879 |
| 14 | 110660001 | 110670000 | 0.127424 |
| 14 | 110720001 | 110730000 | 0.089933 |
| 14 | 110730001 | 110740000 | 0.169432 |
| 14 | 111390001 | 111400000 | 0.09887 |
| 14 | 111400001 | 111410000 | 0.117295 |
| 14 | 111440001 | 111450000 | 0.112796 |
| 14 | 111760001 | 111770000 | 0.096612 |
| 14 | 112240001 | 112250000 | 0.137687 |
| 14 | 112890001 | 112900000 | 0.091952 |
| 14 | 112990001 | 113000000 | 0.108744 |
| 14 | 113120001 | 113130000 | 0.129594 |
| 14 | 113130001 | 113140000 | 0.159295 |
| 14 | 113140001 | 113150000 | 0.169111 |
| 14 | 113160001 | 113170000 | 0.127647 |
| 14 | 113170001 | 113180000 | 0.158939 |
| 14 | 114340001 | 114350000 | 0.090857 |
| 14 | 120430001 | 120440000 | 0.112215 |
| 14 | 130250001 | 130260000 | 0.117743 |
| 14 | 130260001 | 130270000 | 0.090531 |
| 15 | 11530001 | 11540000 | 0.115298 |
| 15 | 21500001 | 21510000 | 0.119557 |
| 15 | 25100001 | 25110000 | 0.162646 |
| 15 | 25140001 | 25150000 | 0.109835 |
| 15 | 25440001 | 25450000 | 0.113495 |
| 15 | 25460001 | 25470000 | 0.130906 |
| 15 | 37830001 | 37840000 | 0.127891 |
| 15 | 37840001 | 37850000 | 0.141881 |
| 15 | 40760001 | 40770000 | 0.127558 |
| 15 | 42330001 | 42340000 | 0.112292 |
| 15 | 42350001 | 42360000 | 0.122054 |
| 15 | 42390001 | 42400000 | 0.195889 |
| 15 | 42710001 | 42720000 | 0.136735 |
| 15 | 42720001 | 42730000 | 0.179567 |
| 15 | 42730001 | 42740000 | 0.127004 |
| 15 | 52460001 | 52470000 | 0.117184 |
| 15 | 52480001 | 52490000 | 0.136319 |
| 15 | 54790001 | 54800000 | 0.129348 |
| 15 | 60660001 | 60670000 | 0.120466 |
| 15 | 93640001 | 93650000 | 0.115129 |
| 15 | 94750001 | 94760000 | 0.17434 |
| 15 | 94760001 | 94770000 | 0.190664 |
| 15 | 94780001 | 94790000 | 0.127223 |
| 15 | 95560001 | 95570000 | 0.113957 |
| 15 | 98760001 | 98770000 | 0.113699 |
| 15 | 98980001 | 98990000 | 0.114014 |
| 15 | 107190001 | 107200000 | 0.114459 |
| 15 | 142730001 | 142740000 | 0.136914 |
| 15 | 148620001 | 148630000 | 0.131924 |
| 15 | 153310001 | 153320000 | 0.139227 |
| 15 | 153320001 | 153330000 | 0.125175 |
| 15 | 153790001 | 153800000 | 0.132104 |
| 16 | 4990001 | 5000000 | 0.081833 |
| 16 | 7270001 | 7280000 | 0.074079 |
| 16 | 7280001 | 7290000 | 0.076238 |
| 16 | 7590001 | 7600000 | 0.084042 |
| 16 | 8980001 | 8990000 | 0.072879 |
| 16 | 8990001 | 9000000 | 0.086182 |
| 16 | 21050001 | 21060000 | 0.090477 |
| 16 | 21590001 | 21600000 | 0.084227 |
| 16 | 24560001 | 24570000 | 0.09031 |
| 16 | 24570001 | 24580000 | 0.1002 |
| 16 | 28220001 | 28230000 | 0.110164 |
| 16 | 39640001 | 39650000 | 0.069066 |
| 16 | 39970001 | 39980000 | 0.073386 |
| 16 | 59050001 | 59060000 | 0.096079 |
| 16 | 65420001 | 65430000 | 0.080934 |
| 16 | 65520001 | 65530000 | 0.072429 |
| 16 | 70940001 | 70950000 | 0.071857 |
| 16 | 71180001 | 71190000 | 0.076733 |
| 16 | 73940001 | 73950000 | 0.077141 |
| 16 | 74290001 | 74300000 | 0.115966 |
| 16 | 75550001 | 75560000 | 0.104427 |
| 16 | 75840001 | 75850000 | 0.073897 |
| 16 | 75850001 | 75860000 | 0.092572 |
| 16 | 75880001 | 75890000 | 0.07616 |
| 16 | 75990001 | 76000000 | 0.116733 |
| 16 | 76000001 | 76010000 | 0.132294 |
| 16 | 76010001 | 76020000 | 0.085721 |
| 16 | 81390001 | 81400000 | 0.08123 |
| 16 | 82320001 | 82330000 | 0.080458 |
| 16 | 83180001 | 83190000 | 0.080514 |
| 16 | 83190001 | 83200000 | 0.080571 |
| 16 | 83200001 | 83210000 | 0.098602 |
| 16 | 83220001 | 83230000 | 0.109226 |
| 16 | 83230001 | 83240000 | 0.10858 |
| 16 | 83240001 | 83250000 | 0.113808 |
| 16 | 83270001 | 83280000 | 0.092823 |
| 16 | 83320001 | 83330000 | 0.071125 |
| 16 | 86310001 | 86320000 | 0.089313 |
| 16 | 86320001 | 86330000 | 0.079331 |
| 16 | 86330001 | 86340000 | 0.077162 |
| 16 | 86420001 | 86430000 | 0.15265 |
| 17 | 920001 | 930000 | 0.09237 |
| 17 | 16990001 | 17000000 | 0.087407 |
| 17 | 21320001 | 21330000 | 0.107785 |
| 17 | 23840001 | 23850000 | 0.090058 |
| 17 | 23860001 | 23870000 | 0.118895 |
| 17 | 26830001 | 26840000 | 0.089447 |
| 17 | 27880001 | 27890000 | 0.118808 |
| 17 | 27890001 | 27900000 | 0.120914 |
| 17 | 28550001 | 28560000 | 0.087045 |
| 17 | 30200001 | 30210000 | 0.206324 |
| 17 | 30210001 | 30220000 | 0.102396 |
| 17 | 33060001 | 33070000 | 0.137218 |
| 17 | 33070001 | 33080000 | 0.136989 |
| 17 | 33400001 | 33410000 | 0.11859 |
| 17 | 33420001 | 33430000 | 0.098106 |
| 17 | 34840001 | 34850000 | 0.101937 |
| 17 | 36950001 | 36960000 | 0.107256 |
| 17 | 41470001 | 41480000 | 0.10685 |
| 17 | 41530001 | 41540000 | 0.11197 |
| 17 | 41540001 | 41550000 | 0.120997 |
| 17 | 41590001 | 41600000 | 0.090715 |
| 17 | 50270001 | 50280000 | 0.101795 |
| 17 | 57330001 | 57340000 | 0.095515 |
| 17 | 66950001 | 66960000 | 0.093724 |
| 17 | 68440001 | 68450000 | 0.103091 |
| 18 | 5800001 | 5810000 | 0.079584 |
| 18 | 10170001 | 10180000 | 0.099275 |
| 18 | 10950001 | 10960000 | 0.080016 |
| 18 | 15280001 | 15290000 | 0.069299 |
| 18 | 15300001 | 15310000 | 0.072067 |
| 18 | 27070001 | 27080000 | 0.084233 |
| 18 | 28720001 | 28730000 | 0.067091 |
| 18 | 28850001 | 28860000 | 0.122973 |
| 18 | 28860001 | 28870000 | 0.101146 |
| 18 | 42950001 | 42960000 | 0.087425 |
| 18 | 46200001 | 46210000 | 0.078916 |
| 18 | 47890001 | 47900000 | 0.074811 |
| 18 | 56460001 | 56470000 | 0.098772 |
| 18 | 56840001 | 56850000 | 0.083638 |
| 18 | 56970001 | 56980000 | 0.070553 |
| 18 | 57030001 | 57040000 | 0.101801 |
| 18 | 57240001 | 57250000 | 0.075788 |
| 18 | 57250001 | 57260000 | 0.12096 |
| 18 | 57260001 | 57270000 | 0.080993 |
| 18 | 60510001 | 60520000 | 0.081585 |

**Supplementary Table S6 | Genomic signals identified by XP-EHH- and IBD-based screening of SWP regulating loci.**

| CHR | Star position | End position | Genes |
| --- | --- | --- | --- |
| chr1 | 254,553,390 | 254,833,790 | *RORB, TRPM6* |
| chr1 | 308,659,813 | 308,704,725 | *BLG, GLT6D1, LCN9* |
| chr1 | 308,703,493 | 308,715,241 | *UCAL-P19* |
| chr4 | 87,884,137 | 90,090,712 | *ATP1B1, BLZF1, C1ORF112, C1orf114,DPT， F5, GORAB, KIFAP3, METTL11B, METTL18, NME7, SLC9A1, PRRX1, SCYL3, SELE, SELL, SELP* |
| chr4 | 91,596,071 | 91,597,544 | *GPA33* |
| chr6 | 7,303,4245 | 73,296,649 | *CDA, DDOST, EIF4G3, HP1BP3, KIF17, PINK1, SH2D5* |
| chr6 | 143,323,160 | 143,514,822 | *C1orf168* |
| chr9 | 39,275,026 | 40,177,218 | *AASDHPPT, ALKBH8, CWF19L2, GRIA4, KBTBD3, KIAA1826* |
| chr9 | 107,043,002 | 107,107,733 | *SEMA3E* |
| chr15 | 31,711,773 | 31,788,518 | *CNTNAP5* |
| chr15 | 75,325,202 | 75,997,864 | *PSMD14, SLC4A10, TANK, TBR1* |
| chr15 | 120,333,794 | 120,624,951 | *PARD3B* |
| chr18 | 46,309,276 | 46,310,717 | *PAC1* |

**Supplementary Table S7 | 18 tagged SNPs for 18 candidate genes used in an association analysis of the SWP phenotype.**

| CHR | Position | Gene | Mutation |
| --- | --- | --- | --- |
| chr1 | 229,151,265 | *CNTLN* | G > T |
| chr4 | 39,532,637 | *RNF19A* | C > T |
| chr4 | 40,901,362 | *OSR2* | C > T |
| chr5 | 53,130,878 | *SOX5* | T > A |
| chr5 | 81,642,632 | *SENP1* | A > G |
| chr6 | 73,118,955 | *PINK1* | C > T |
| chr7 | 54,807,944 | *ARNT2* | C > T |
| chr8 | 53,930,374 | *FSTL5* | A > C |
| chr11 | 54,762,085 | *EDNRB* | G > A |
| chr11 | 71,412,807 | *ABCC4* | T > C |
| chr11 | 81,925,800 | *EDA2R* | T > C |
| chr12 | 34,761,833 | *MSI2* | T > C |
| chr13 | 140,229,419 | *OPA1* | C > A |
| chr13 | 193,328,223 | *TMPRSS15* | A > C |
| chr13 | 213,659,304 | *DSCAM* | C > G |
| chr14 | 70,198,568 | *C10orf107* | C > T |
| chr15 | 11,815,900 | *LRP1B* | G > A |
| chr15 | 92,243,930 | *TVP23C* | C > T |

**Supplementary Table S8 | SNPs upstream of the *EDNRB* on chromosome 11 highly differentiated between SWP and black DSE pigs.**

| Position | Alleles | Region | Gene on left side | Position | Gene on right side | Position |
| --- | --- | --- | --- | --- | --- | --- |
| 54,750,820 | G/T | Intergenic region | *EDNRB* | 54,718,024 | *POU4F1* | 55,419,671 |
| 54,750,836 | G/A | Intergenic region | *EDNRB* | 54,718,024 | *POU4F1* | 55,419,671 |
| 54,750,859 | G/A | Intergenic region | *EDNRB* | 54,718,024 | *POU4F1* | 55,419,671 |
| 54,751,021 | G/A | Intergenic region | *EDNRB* | 54,718,024 | *POU4F1* | 55,419,671 |
| 54,751,080 | A/T | Intergenic region | *EDNRB* | 54,718,024 | *POU4F1* | 55,419,671 |
| 54,751,096 | G/A | Intergenic region | *EDNRB* | 54,718,024 | *POU4F1* | 55,419,671 |
| 54,751,305 | T/C | Intergenic region | *EDNRB* | 54,718,024 | *POU4F1* | 55,419,671 |
| 54,751,367 | A/G | Intergenic region | *EDNRB* | 54,718,024 | *POU4F1* | 55,419,671 |
| 54,751,382 | T/A | Intergenic region | *EDNRB* | 54,718,024 | *POU4F1* | 55,419,671 |
| 54,751,400 | C/G | Intergenic region | *EDNRB* | 54,718,024 | *POU4F1* | 55,419,671 |
| 54,751,482 | C/T | Intergenic region | *EDNRB* | 54,718,024 | *POU4F1* | 55,419,671 |
| 54,752,047 | G/C | Intergenic region | *EDNRB* | 54,718,024 | *POU4F1* | 55,419,671 |
| 54,752,085 | G/A | Intergenic region | *EDNRB* | 54,718,024 | *POU4F1* | 55,419,671 |
| 54,753,070 | G/A | Intergenic region | *EDNRB* | 54,718,024 | *POU4F1* | 55,419,671 |
| 54,753,075 | C/T | Intergenic region | *EDNRB* | 54,718,024 | *POU4F1* | 55,419,671 |
| 54,753,301 | C/A | Intergenic region | *EDNRB* | 54,718,024 | *POU4F1* | 55,419,671 |
| 54,753,386 | A/T | Intergenic region | *EDNRB* | 54,718,024 | *POU4F1* | 55,419,671 |
| 54,753,890 | C/T | Intergenic region | *EDNRB* | 54,718,024 | *POU4F1* | 55,419,671 |
| 54,753,914 | C/T | Intergenic region | *EDNRB* | 54,718,024 | *POU4F1* | 55,419,671 |
| 54,754,348 | G/A | Intergenic region | *EDNRB* | 54,718,024 | *POU4F1* | 55,419,671 |
| 54,754,518 | A/T | Intergenic region | *EDNRB* | 54,718,024 | *POU4F1* | 55,419,671 |
| 54,754,627 | T/C | Intergenic region | *EDNRB* | 54,718,024 | *POU4F1* | 55,419,671 |
| 54,754,650 | G/C | Intergenic region | *EDNRB* | 54,718,024 | *POU4F1* | 55,419,671 |
| 54,755,214 | G/A | Intergenic region | *EDNRB* | 54,718,024 | *POU4F1* | 55,419,671 |
| 54,755,335 | C/A | Intergenic region | *EDNRB* | 54,718,024 | *POU4F1* | 55,419,671 |
| 54,755,618 | G/A | Intergenic region | *EDNRB* | 54,718,024 | *POU4F1* | 55,419,671 |
| 54,755,665 | T/A | Intergenic region | *EDNRB* | 54,718,024 | *POU4F1* | 55,419,671 |
| 54,755,912 | T/C | Intergenic region | *EDNRB* | 54,718,024 | *POU4F1* | 55,419,671 |
| 54,756,045 | G/A | Intergenic region | *EDNRB* | 54,718,024 | *POU4F1* | 55,419,671 |
| 54,757,033 | T/C | Intergenic region | *EDNRB* | 54,718,024 | *POU4F1* | 55,419,671 |
| 54,757,039 | T/C | Intergenic region | *EDNRB* | 54,718,024 | *POU4F1* | 55,419,671 |
| 54,757,058 | G/A | Intergenic region | *EDNRB* | 54,718,024 | *POU4F1* | 55,419,671 |
| 54,757,869 | C/T | Intergenic region | *EDNRB* | 54,718,024 | *POU4F1* | 55,419,671 |
| 54,758,748 | T/G | Intergenic region | *EDNRB* | 54,718,024 | *POU4F1* | 55,419,671 |
| 54,758,754 | G/A | Intergenic region | *EDNRB* | 54,718,024 | *POU4F1* | 55,419,671 |
| 54,760,228 | C/T | Intergenic region | *EDNRB* | 54,718,024 | *POU4F1* | 55,419,671 |
| 54,760,351 | T/G | Intergenic region | *EDNRB* | 54,718,024 | *POU4F1* | 55,419,671 |
| 54,760,392 | T/G | Intergenic region | *EDNRB* | 54,718,024 | *POU4F1* | 55,419,671 |
| 54,760,438 | G/A | Intergenic region | *EDNRB* | 54,718,024 | *POU4F1* | 55,419,671 |
| 54,760,498 | G/A | Intergenic region | *EDNRB* | 54,718,024 | *POU4F1* | 55,419,671 |
| 54,760,508 | A/C | Intergenic region | *EDNRB* | 54,718,024 | *POU4F1* | 55,419,671 |
| 54,760,514 | G/A | Intergenic region | *EDNRB* | 54,718,024 | *POU4F1* | 55,419,671 |
| 54,760,598 | A/G | Intergenic region | *EDNRB* | 54,718,024 | *POU4F1* | 55,419,671 |
| 54,760,613 | C/T | Intergenic region | *EDNRB* | 54,718,024 | *POU4F1* | 55,419,671 |
| 54,760,629 | A/T | Intergenic region | *EDNRB* | 54,718,024 | *POU4F1* | 55,419,671 |
| 54,760,981 | G/T | Intergenic region | *EDNRB* | 54,718,024 | *POU4F1* | 55,419,671 |
| 54,761,126 | T/C | Intergenic region | *EDNRB* | 54,718,024 | *POU4F1* | 55,419,671 |
| 54,762,085 | G/A | Intergenic region | *EDNRB* | 54,718,024 | *POU4F1* | 55,419,671 |
| 54,762,376 | C/T | Intergenic region | *EDNRB* | 54,718,024 | *POU4F1* | 55,419,671 |
| 54,762,554 | A/G | Intergenic region | *EDNRB* | 54,718,024 | *POU4F1* | 55,419,671 |
| 54,762,782 | G/A | Intergenic region | *EDNRB* | 54,718,024 | *POU4F1* | 55,419,671 |
| 54,762,809 | C/A | Intergenic region | *EDNRB* | 54,718,024 | *POU4F1* | 55,419,671 |
| 54,764,752 | A/G | Intergenic region | *EDNRB* | 54,718,024 | *POU4F1* | 55,419,671 |
| 54,765,124 | C/T | Intergenic region | *EDNRB* | 54,718,024 | *POU4F1* | 55,419,671 |
| 54,766,113 | T/C | Intergenic region | *EDNRB* | 54,718,024 | *POU4F1* | 55,419,671 |
| 54,766,188 | T/C | Intergenic region | *EDNRB* | 54,718,024 | *POU4F1* | 55,419,671 |
| 54,766,623 | C/T | Intergenic region | *EDNRB* | 54,718,024 | *POU4F1* | 55,419,671 |
| 54,766,724 | G/A | Intergenic region | *EDNRB* | 54,718,024 | *POU4F1* | 55,419,671 |
| 54,766,758 | G/A | Intergenic region | *EDNRB* | 54,718,024 | *POU4F1* | 55,419,671 |
| 54,766,782 | G/A | Intergenic region | *EDNRB* | 54,718,024 | *POU4F1* | 55,419,671 |
| 54,767,003 | C/A | Intergenic region | *EDNRB* | 54,718,024 | *POU4F1* | 55,419,671 |
| 54,768,185 | T/C | Intergenic region | *EDNRB* | 54,718,024 | *POU4F1* | 55,419,671 |
| 54,768,247 | G/A | Intergenic region | *EDNRB* | 54,718,024 | *POU4F1* | 55,419,671 |
| 54,768,587 | A/G | Intergenic region | *EDNRB* | 54,718,024 | *POU4F1* | 55,419,671 |
| 54,768,622 | C/G | Intergenic region | *EDNRB* | 54,718,024 | *POU4F1* | 55,419,671 |
| 54,768,644 | A/C | Intergenic region | *EDNRB* | 54,718,024 | *POU4F1* | 55,419,671 |
| 54,768,707 | G/C | Intergenic region | *EDNRB* | 54,718,024 | *POU4F1* | 55,419,671 |
| 54,768,740 | T/C | Intergenic region | *EDNRB* | 54,718,024 | *POU4F1* | 55,419,671 |
| 54,768,742 | C/T | Intergenic region | *EDNRB* | 54,718,024 | *POU4F1* | 55,419,671 |
| 54,768,777 | G/A | Intergenic region | *EDNRB* | 54,718,024 | *POU4F1* | 55,419,671 |
| 54,768,780 | G/A | Intergenic region | *EDNRB* | 54,718,024 | *POU4F1* | 55,419,671 |
| 54,768,793 | T/C | Intergenic region | *EDNRB* | 54,718,024 | *POU4F1* | 55,419,671 |
| 54,768,815 | C/T | Intergenic region | *EDNRB* | 54,718,024 | *POU4F1* | 55,419,671 |
| 54,768,904 | A/G | Intergenic region | *EDNRB* | 54,718,024 | *POU4F1* | 55,419,671 |
| 54,768,961 | T/C | Intergenic region | *EDNRB* | 54,718,024 | *POU4F1* | 55,419,671 |
| 54,768,995 | T/C | Intergenic region | *EDNRB* | 54,718,024 | *POU4F1* | 55,419,671 |
| 54,768,996 | G/A | Intergenic region | *EDNRB* | 54,718,024 | *POU4F1* | 55,419,671 |
| 54,769,029 | A/G | Intergenic region | *EDNRB* | 54,718,024 | *POU4F1* | 55,419,671 |
| 54,769,051 | G/A | Intergenic region | *EDNRB* | 54,718,024 | *POU4F1* | 55,419,671 |
| 54,769,064 | G/A | Intergenic region | *EDNRB* | 54,718,024 | *POU4F1* | 55,419,671 |
| 54,769,069 | G/A | Intergenic region | *EDNRB* | 54,718,024 | *POU4F1* | 55,419,671 |
| 54,769,073 | G/A | Intergenic region | *EDNRB* | 54,718,024 | *POU4F1* | 55,419,671 |
| 54,769,081 | A/G | Intergenic region | *EDNRB* | 54,718,024 | *POU4F1* | 55,419,671 |
| 54,769,108 | C/T | Intergenic region | *EDNRB* | 54,718,024 | *POU4F1* | 55,419,671 |
| 54,769,123 | G/T | Intergenic region | *EDNRB* | 54,718,024 | *POU4F1* | 55,419,671 |
| 54,769,204 | T/C | Intergenic region | *EDNRB* | 54,718,024 | *POU4F1* | 55,419,671 |
| 54,769,314 | A/T | Intergenic region | *EDNRB* | 54,718,024 | *POU4F1* | 55,419,671 |
| 54,769,363 | G/A | Intergenic region | *EDNRB* | 54,718,024 | *POU4F1* | 55,419,671 |
| 54,769,400 | G/A | Intergenic region | *EDNRB* | 54,718,024 | *POU4F1* | 55,419,671 |
| 54,769,424 | G/A | Intergenic region | *EDNRB* | 54,718,024 | *POU4F1* | 55,419,671 |
| 54,769,730 | C/T | Intergenic region | *EDNRB* | 54,718,024 | *POU4F1* | 55,419,671 |
| 54,769,742 | C/T | Intergenic region | *EDNRB* | 54,718,024 | *POU4F1* | 55,419,671 |
| 54,770,026 | T/C | Intergenic region | *EDNRB* | 54,718,024 | *POU4F1* | 55,419,671 |
| 54,770,041 | C/T | Intergenic region | *EDNRB* | 54,718,024 | *POU4F1* | 55,419,671 |
| 54,770,186 | C/G | Intergenic region | *EDNRB* | 54,718,024 | *POU4F1* | 55,419,671 |
| 54,770,500 | C/A | Intergenic region | *EDNRB* | 54,718,024 | *POU4F1* | 55,419,671 |
| 54,770,533 | G/A | Intergenic region | *EDNRB* | 54,718,024 | *POU4F1* | 55,419,671 |
| 54,771,347 | G/A | Intergenic region | *EDNRB* | 54,718,024 | *POU4F1* | 55,419,671 |
| 54,771,881 | G/A | Intergenic region | *EDNRB* | 54,718,024 | *POU4F1* | 55,419,671 |
| 54,771,926 | C/G | Intergenic region | *EDNRB* | 54,718,024 | *POU4F1* | 55,419,671 |
| 54,772,188 | G/A | Intergenic region | *EDNRB* | 54,718,024 | *POU4F1* | 55,419,671 |
| 54,772,210 | G/A | Intergenic region | *EDNRB* | 54,718,024 | *POU4F1* | 55,419,671 |
| 54,772,301 | T/C | Intergenic region | *EDNRB* | 54,718,024 | *POU4F1* | 55,419,671 |
| 54,772,319 | A/G | Intergenic region | *EDNRB* | 54,718,024 | *POU4F1* | 55,419,671 |
| 54,772,379 | G/C | Intergenic region | *EDNRB* | 54,718,024 | *POU4F1* | 55,419,671 |
| 54,772,478 | C/T | Intergenic region | *EDNRB* | 54,718,024 | *POU4F1* | 55,419,671 |
| 54,772,570 | C/T | Intergenic region | *EDNRB* | 54,718,024 | *POU4F1* | 55,419,671 |
| 54,772,619 | C/T | Intergenic region | *EDNRB* | 54,718,024 | *POU4F1* | 55,419,671 |
| 54,772,899 | G/C | Intergenic region | *EDNRB* | 54,718,024 | *POU4F1* | 55,419,671 |
| 54,773,270 | G/A | Intergenic region | *EDNRB* | 54,718,024 | *POU4F1* | 55,419,671 |
| 54,773,366 | T/C | Intergenic region | *EDNRB* | 54,718,024 | *POU4F1* | 55,419,671 |
| 54,773,428 | C/T | Intergenic region | *EDNRB* | 54,718,024 | *POU4F1* | 55,419,671 |
| 54,773,721 | G/C | Intergenic region | *EDNRB* | 54,718,024 | *POU4F1* | 55,419,671 |
| 54,773,728 | C/T | Intergenic region | *EDNRB* | 54,718,024 | *POU4F1* | 55,419,671 |
| 54,773,766 | C/T | Intergenic region | *EDNRB* | 54,718,024 | *POU4F1* | 55,419,671 |
| 54,773,774 | C/T | Intergenic region | *EDNRB* | 54,718,024 | *POU4F1* | 55,419,671 |
| 54,773,809 | C/T | Intergenic region | *EDNRB* | 54,718,024 | *POU4F1* | 55,419,671 |
| 54,773,847 | G/A | Intergenic region | *EDNRB* | 54,718,024 | *POU4F1* | 55,419,671 |
| 54,773,978 | A/T | Intergenic region | *EDNRB* | 54,718,024 | *POU4F1* | 55,419,671 |
| 54,774,018 | C/T | Intergenic region | *EDNRB* | 54,718,024 | *POU4F1* | 55,419,671 |
| 54,774,048 | G/A | Intergenic region | *EDNRB* | 54,718,024 | *POU4F1* | 55,419,671 |
| 54,774,293 | A/T | Intergenic region | *EDNRB* | 54,718,024 | *POU4F1* | 55,419,671 |
| 54,775,083 | A/G | Intergenic region | *EDNRB* | 54,718,024 | *POU4F1* | 55,419,671 |
| 54,776,292 | G/A | Intergenic region | *EDNRB* | 54,718,024 | *POU4F1* | 55,419,671 |
| 54,777,176 | T/C | Intergenic region | *EDNRB* | 54,718,024 | *POU4F1* | 55,419,671 |
| 54,777,231 | C/T | Intergenic region | *EDNRB* | 54,718,024 | *POU4F1* | 55,419,671 |
| 54,777,295 | T/C | Intergenic region | *EDNRB* | 54,718,024 | *POU4F1* | 55,419,671 |
| 54,778,140 | T/G | Intergenic region | *EDNRB* | 54,718,024 | *POU4F1* | 55,419,671 |
| 54,828,755 | C/T | Intergenic region | *EDNRB* | 54,718,024 | *POU4F1* | 55,419,671 |
| 54,829,348 | G/A | Intergenic region | *EDNRB* | 54,718,024 | *POU4F1* | 55,419,671 |
| 54,829,740 | G/A | Intergenic region | *EDNRB* | 54,718,024 | *POU4F1* | 55,419,671 |
| 54,830,425 | C/T | Intergenic region | *EDNRB* | 54,718,024 | *POU4F1* | 55,419,671 |
| 54,831,118 | A/T | Intergenic region | *EDNRB* | 54,718,024 | *POU4F1* | 55,419,671 |
| 54,831,646 | C/T | Intergenic region | *EDNRB* | 54,718,024 | *POU4F1* | 55,419,671 |
| 54,831,873 | C/T | Intergenic region | *EDNRB* | 54,718,024 | *POU4F1* | 55,419,671 |
| 54,831,925 | C/A | Intergenic region | *EDNRB* | 54,718,024 | *POU4F1* | 55,419,671 |
| 54,832,199 | G/A | Intergenic region | *EDNRB* | 54,718,024 | *POU4F1* | 55,419,671 |
| 54,832,568 | C/T | Intergenic region | *EDNRB* | 54,718,024 | *POU4F1* | 55,419,671 |
| 54,832,589 | C/T | Intergenic region | *EDNRB* | 54,718,024 | *POU4F1* | 55,419,671 |
| 54,832,764 | T/G | Intergenic region | *EDNRB* | 54,718,024 | *POU4F1* | 55,419,671 |
| 54,833,055 | G/A | Intergenic region | *EDNRB* | 54,718,024 | *POU4F1* | 55,419,671 |
| 54,833,088 | G/A | Intergenic region | *EDNRB* | 54,718,024 | *POU4F1* | 55,419,671 |
| 54,833,200 | C/T | Intergenic region | *EDNRB* | 54,718,024 | *POU4F1* | 55,419,671 |
| 54,833,262 | C/T | Intergenic region | *EDNRB* | 54,718,024 | *POU4F1* | 55,419,671 |
| 54,833,300 | G/A | Intergenic region | *EDNRB* | 54,718,024 | *POU4F1* | 55,419,671 |
| 54,833,528 | C/T | Intergenic region | *EDNRB* | 54,718,024 | *POU4F1* | 55,419,671 |
| 54,833,631 | T/C | Intergenic region | *EDNRB* | 54,718,024 | *POU4F1* | 55,419,671 |
| 54,833,706 | C/T | Intergenic region | *EDNRB* | 54,718,024 | *POU4F1* | 55,419,671 |
| 54,833,710 | T/A | Intergenic region | *EDNRB* | 54,718,024 | *POU4F1* | 55,419,671 |
| 54,833,812 | G/A | Intergenic region | *EDNRB* | 54,718,024 | *POU4F1* | 55,419,671 |
| 54,833,861 | T/C | Intergenic region | *EDNRB* | 54,718,024 | *POU4F1* | 55,419,671 |
| 54,834,130 | C/T | Intergenic region | *EDNRB* | 54,718,024 | *POU4F1* | 55,419,671 |
| 54,834,854 | T/A | Intergenic region | *EDNRB* | 54,718,024 | *POU4F1* | 55,419,671 |
| 54,834,961 | G/A | Intergenic region | *EDNRB* | 54,718,024 | *POU4F1* | 55,419,671 |
| 54,835,042 | C/A | Intergenic region | *EDNRB* | 54,718,024 | *POU4F1* | 55,419,671 |
| 54,835,615 | C/T | Intergenic region | *EDNRB* | 54,718,024 | *POU4F1* | 55,419,671 |
| 54,835,766 | C/T | Intergenic region | *EDNRB* | 54,718,024 | *POU4F1* | 55,419,671 |
| 54,836,236 | G/A | Intergenic region | *EDNRB* | 54,718,024 | *POU4F1* | 55,419,671 |
| 54,837,948 | A/G | Intergenic region | *EDNRB* | 54,718,024 | *POU4F1* | 55,419,671 |
| 54,837,959 | A/G | Intergenic region | *EDNRB* | 54,718,024 | *POU4F1* | 55,419,671 |
| 54,838,195 | T/C | Intergenic region | *EDNRB* | 54,718,024 | *POU4F1* | 55,419,671 |
| 54,838,247 | T/C | Intergenic region | *EDNRB* | 54,718,024 | *POU4F1* | 55,419,671 |
| 54,838,286 | T/A | Intergenic region | *EDNRB* | 54,718,024 | *POU4F1* | 55,419,671 |
| 54,839,056 | G/A | Intergenic region | *EDNRB* | 54,718,024 | *POU4F1* | 55,419,671 |
| 54,839,411 | G/A | Intergenic region | *EDNRB* | 54,718,024 | *POU4F1* | 55,419,671 |
| 54,839,866 | A/G | Intergenic region | *EDNRB* | 54,718,024 | *POU4F1* | 55,419,671 |
| 54,840,236 | G/A | Intergenic region | *EDNRB* | 54,718,024 | *POU4F1* | 55,419,671 |
| 54,841,719 | T/C | Intergenic region | *EDNRB* | 54,718,024 | *POU4F1* | 55,419,671 |
| 54,841,732 | T/C | Intergenic region | *EDNRB* | 54,718,024 | *POU4F1* | 55,419,671 |
| 54,842,071 | G/A | Intergenic region | *EDNRB* | 54,718,024 | *POU4F1* | 55,419,671 |
| 54,842,116 | G/A | Intergenic region | *EDNRB* | 54,718,024 | *POU4F1* | 55,419,671 |
| 54,843,866 | A/C | Intergenic region | *EDNRB* | 54,718,024 | *POU4F1* | 55,419,671 |
| 54,844,691 | G/T | Intergenic region | *EDNRB* | 54,718,024 | *POU4F1* | 55,419,671 |
| 54,844,825 | C/A | Intergenic region | *EDNRB* | 54,718,024 | *POU4F1* | 55,419,671 |
| 54,846,141 | C/G | Intergenic region | *EDNRB* | 54,718,024 | *POU4F1* | 55,419,671 |
| 54,846,522 | G/T | Intergenic region | *EDNRB* | 54,718,024 | *POU4F1* | 55,419,671 |
| 54,846,828 | C/T | Intergenic region | *EDNRB* | 54,718,024 | *POU4F1* | 55,419,671 |
| 54,846,836 | G/A | Intergenic region | *EDNRB* | 54,718,024 | *POU4F1* | 55,419,671 |
| 54,849,244 | A/G | Intergenic region | *EDNRB* | 54,718,024 | *POU4F1* | 55,419,671 |
